# How packaging cues shape purchase intentions for intangible cultural heritage foods: evidence from heritage labels and storytelling

**DOI:** 10.3389/fpsyg.2026.1930687

**Published:** 2026-09-23

**Authors:** Mingzhu Yu, Xuan Qin

**Affiliations:** School of Art and Design, Ma’anshan University, Ma’anshan, Anhui, China

**Keywords:** cultural identity, intangible cultural heritage label, purchase intention, purchase motivation, storytelling

## Abstract

Intangible cultural heritage (ICH) food embodies local history, traditional craftsmanship, and cultural values. However, these attributes are often difficult for consumers to evaluate directly and therefore need to be communicated through packaging information. Drawing primarily on cue utilization theory and incorporating construal level theory, this study examines how storytelling and ICH labels influence consumers’ purchase intention and how purchase motivation shapes the effects of these two packaging cues. Using Caishiji dried tofu, a Chinese ICH food, as the research context, the study employed a 2 × 2 × 2 between-subjects experimental design in which storytelling, the presence of an ICH label, and purchase motivation were manipulated. A total of 428 valid responses were obtained. The study further examined the mediating role of consumers’ cultural identity with the ICH food in the relationship between packaging cues and purchase intention. The results showed that storytelling significantly enhanced consumers’ cultural identity with the ICH food and their purchase intention, whereas the ICH label did not produce a consistent independent effect. In the self-purchase context, storytelling alone generated a positive effect. In the gift-giving context, the joint presentation of storytelling and the ICH label produced a stronger positive effect. The conditional indirect-effect analysis indicated that storytelling was consistently positively associated with purchase intention through cultural identity, whereas the indirect effect of the ICH label depended on whether storytelling was present. However, purchase motivation did not significantly moderate the overall indirect effects. Because cultural identity and purchase intention were measured concurrently, the proposed mediating relationships require further validation in future research. This study demonstrates that packaging cues for ICH food do not universally produce consistent positive effects. Rather, their effectiveness depends on how the cues are presented and combined, as well as on the purchase context. These findings provide a theoretical basis for the context-sensitive design of packaging communication strategies for ICH food.

## 1 Introduction

As a distinctive type of food deeply embedded in local culture and traditional craftsmanship, ICH food typically possesses a clear historical origin, relatively stable handmade production techniques, and cultural meanings transmitted across generations. It is not only a tradable commodity, but also a cultural carrier that continues to be practiced and reproduced (Lee et al., 2018; Mykletun and Gyimóthy, 2010). With the growing awareness of intangible cultural heritage protection and the development of the cultural consumption market, an increasing number of ICH food products that once existed primarily in everyday local life have been reintroduced into modern systems of communication, branding, and consumption (Peštek and Činjarević, 2014). In this process, packaging has gradually become the most direct and central cultural interface through which consumers encounter ICH food, as the traditions and values embodied in the product are often first perceived and understood through its packaging (Wang et al., 2025).

Storytelling and certification labels represent two common types of packaging cues. Storytelling organizes cultural information around characters, temporal sequences, and events, thereby rendering abstract traditions more concrete and comprehensible and facilitating consumers’ emotional engagement (Gasparin et al., 2022; Wang et al., 2025). Labels, by contrast, communicate information about origin, certification, or authenticity through concise symbolic forms, helping consumers reduce uncertainty in product evaluation (Moreira et al., 2021; Truong et al., 2021).

Previous research has compared the effects of storytelling and geographical indication labels on consumers’ evaluations of food products in social media communication (Barretta et al., 2025). However, geographical indications and other certification labels primarily assist consumers in evaluating products by signaling geographical origin, quality standards, or authenticity. By contrast, the central function of an ICH label is to indicate the connection between a product or brand and a specific ICH element and its associated living cultural tradition. As a particular type of heritage label, an ICH label further emphasizes knowledge, skills, and cultural expressions that are recognized by communities, transmitted across generations, and continuously practiced, rather than merely indicating a general association between a product and a certain form of heritage (Janse, 2023; Qiu and Zuo, 2023; Huang et al., 2025). Consequently, findings concerning general certification labels cannot be directly applied to explain the effects of ICH labels. Because the practices, knowledge, and cultural expressions embodied in ICH food are often complex, consumers’ recognition of an ICH label does not necessarily mean that they understand the specific cultural content carried by the product. This distinction further underscores the need to examine the independent and combined effects of ICH labels and storytelling.

Nevertheless, three interrelated issues remain unresolved in the existing literature. First, storytelling and ICH labels represent qualitatively distinct cultural cues, yet how they independently and jointly shape consumer judgments has not been systematically explained. Second, the underlying psychological process through which cultural information on packaging influences purchase intention remains unclear. Third, the boundary conditions of cultural cues and the reasons why their effects vary with consumers’ decision goals require further investigation.

Cue utilization theory provides an overarching framework for addressing these issues. Consumers form judgments based on the credibility and interpretability of available cues, as well as their relevance to the current decision goal. Moreover, the effect of one cue may be influenced by other cues presented simultaneously. However, existing cue-based research has primarily examined how extrinsic cues shape perceptions of quality, origin, or authenticity, while offering limited explanations of the psychological process through which ICH-related cultural information enters purchase decisions. Research on cultural identity has shown that local identity, cultural values, and the cultural embeddedness of traditions can influence consumers’ attitudes toward and purchase intentions for local foods and cultural products. Nevertheless, it has not fully explained how specific packaging cues evoke consumers’ cultural understanding of, emotional connection with, and identification with a particular ICH food. Accordingly, this study conceptualizes cultural identity as a potential psychological mechanism linking external cultural cues to consumers’ purchase responses (Wang et al., 2025).

Moreover, although cue utilization theory suggests that the value of a cue depends on the decision goal, it does not specifically explain why different purchase motivations lead consumers to prioritize cultural cues differently. Construal level theory complements this perspective. Self-purchase and gift-giving involve different levels of emphasis on concrete consumption experiences and cultural-symbolic meanings, which may alter the relative roles of storytelling and ICH labels in consumer decision-making (Kim and Littrell, 2001; Lyu et al., 2022; Zhu et al., 2024). Accordingly, within the framework of cue utilization theory, this study examines the independent and combined effects of storytelling and ICH labels and further investigates the psychological role of cultural identity and the contextual differences arising from purchase motivation. Based on these considerations, the following research questions are proposed:

*RQ1*: How do consumers interpret and integrate the different types of cultural information conveyed by storytelling and ICH labels on ICH food packaging, and what role does consumers’ cultural identity with a specific ICH food play in this process?

*RQ2*: How do different purchase motivations alter the relative diagnostic value of storytelling and ICH labels and, in turn, influence the translation of cultural cues into purchase decisions?

By addressing these research questions, this study advances the existing literature in three main respects. First, it extends cue utilization theory to the context of ICH food packaging by examining how storytelling and ICH labels, as two distinct types of cultural cues, independently and jointly shape consumer judgments. In doing so, the study enriches the explanatory scope of cue utilization theory in food consumption contexts in which cultural attributes cannot be directly observed by consumers. Second, this study introduces consumers’ cultural identity with a specific ICH food as a potential psychological mechanism, thereby providing further insight into the process through which cultural information conveyed on packaging influences purchase intention. It demonstrates that consumers’ responses to ICH food depend not only on whether they recognize the relevant cultural information but also on whether such information facilitates cultural understanding, acceptance, and emotional connection. Third, this study incorporates purchase motivation into the cue utilization process and draws on construal level theory to explain how self-purchase and gift-giving goals alter the relative importance consumers attach to different cultural cues. It thus clarifies the contextual boundaries within which storytelling and ICH labels exert their effects.

## 2 Literature review

### 2.1 ICH food and packaging Cue utilization

As an important component of intangible cultural heritage, ICH food is not merely food for consumption, but a distinctive form of cultural asset that integrates food, place, craftsmanship, and customs into a shared cultural expression (Sjöholm, 2016). Previous studies have defined ICH food as “the sum of the tangible and intangible elements of food culture that are regarded as shared heritage or public resources” (Almansouri et al., 2021).

Compared with ordinary food, the distinctiveness of ICH food lies in the fact that it not only serves sensory and consumption-related functions, but also embodies traditional craftsmanship, local culture, and social symbolic meanings (Peštek and Činjarević, 2014). Fischler (1988) argued that food is an important factor in shaping individuals, identity, culture, and even society, and that it carries meanings unique to a particular culture. Therefore, ICH food is essentially a form of cultural expression that goes beyond physical attributes, condensing narratives, traditional craftsmanship, social customs, and local memory (Lee, 2023; Shahrin and Hussin, 2023; Chukwurah et al., 2025). Accordingly, the historical origins, traditional craftsmanship, and cultural value of ICH food are often difficult to assess directly from the product itself. Consumers therefore commonly rely on packaging and other informational cues to form their perceptions (Liang et al., 2026).

The cultural information associated with ICH food can be incorporated into packaging in different forms. This study focuses on two representative cultural cues: storytelling and ICH labels. Storytelling organizes product information around characters, time, place, and sequences of events, enabling consumers to understand how a tradition developed and why a product carries cultural significance (Woodside, 2010; Fenger et al., 2015). In contrast, an ICH label uses a concise visual identifier to communicate the connection between a product and a particular category of intangible cultural heritage, its status as a transmitted cultural practice, and its official recognition (Qiu and Zuo, 2023; Huang et al., 2025). Cue utilization theory proposes that when consumers are unable to evaluate product attributes directly, they rely on available intrinsic and extrinsic cues to form judgments. Whether a cue influences evaluation depends on its availability, credibility, and diagnostic value for the decision at hand (Olson, 1972). Moreover, the effects of cues are not fixed. The informational form of a cue, the consistency among multiple cues, consumers’ understanding of those cues, and the specific purchase context may all alter the weight assigned to different cues in consumer judgment (Miyazaki et al., 2005). Thus, both storytelling and ICH labels may shape consumer evaluations, but they differ in their informational forms and in the bases through which they exert their effects. Nevertheless, existing research has not sufficiently explained how distinct cultural cues jointly shape consumers’ evaluations of ICH food, and their underlying mechanisms and contextual boundaries remain to be further investigated.

### 2.2 Effects of storytelling and ICH labels on purchase intention

#### 2.2.1 The role and boundary conditions of storytelling

Storytelling has become an important marketing strategy for product promotion (Schrobback et al., 2023). It organizes information about a product’s place of origin, production techniques, and historical background into a coherent narrative context. Such communication helps consumers understand where and how a product is produced and enhances their retention of the relevant information (Gasparin et al., 2022; Lundqvist et al., 2013). Storytelling may therefore further shape consumers’ evaluations of product authenticity and value, as well as their purchase decisions (Woodside, 2010; Cao et al., 2021).

In food consumption contexts, brief stories can help consumers understand a product’s ingredients, geographical origin, and production process, thereby improving their evaluations and choices regarding traditional foods or novel products (Barretta et al., 2025; Fenger et al., 2015). Existing research further indicates that narrative information can enhance consumer preference and purchase intention. Even among consumers with relatively low initial interest, a brief and specific story may strengthen their inclination to purchase the product (Fenger et al., 2015; Dominique-Ferreira et al., 2023).

However, the positive effects of storytelling may not emerge consistently across all contexts. When a story is incongruent with the product’s attributes or packaging format, its persuasive advantage may be weakened (Jaud et al., 2023). Moreover, when narrative content is excessively embellished, lacks authenticity, or conveys an overly explicit commercial intent, consumers may perceive it as manipulative communication, thereby developing skepticism and resistance (Krenz et al., 2025). These findings indicate that the effectiveness of storytelling depends on the congruence between the narrative content and product attributes, the credibility of the information, and the level of difficulty consumers experience in interpreting the narrative.

This tension is particularly relevant in the context of ICH food. On the one hand, ICH food is associated with rich historical origins, personal experiences, and processes of intergenerational transmission, providing suitable content for storytelling. On the other hand, such cultural information is relatively complex, and direct evidence remains limited as to whether its narrative presentation can be consistently translated into purchase intention. Based on prior evidence supporting the effects of storytelling on consumer understanding, preference, and purchase responses, this study proposes the following hypothesis:

*H1*: Compared with a conventional product description, storytelling on ICH food packaging will increase consumers’ purchase intention.

#### 2.2.2 The role and boundary conditions of ICH labels

The origin, quality, and authenticity of food products are often difficult for consumers to observe directly. Packaging labels are therefore commonly used as important extrinsic cues for evaluating product credibility. Prior research has shown that certification labels can strengthen consumers’ perceptions of product authenticity and quality, enhance trust and perceived quality, and subsequently increase purchase intention and willingness to pay a price premium (Atkinson and Rosenthal, 2014; Liu et al., 2020; Wang et al., 2020; Wu et al., 2021).

An ICH label similarly represents a highly condensed extrinsic cue. Within the limited space available on product packaging, it signals a product’s ICH status, enables consumers to identify its traditional and cultural attributes rapidly, and may reduce uncertainty regarding its cultural authenticity by drawing on the authority of the recognizing institution (Huang et al., 2025). An ICH label may therefore improve consumers’ purchase evaluations.

However, the positive effects of labels are not consistently observed. When consumers are unfamiliar with the meaning of a label, the recognition criteria, or the certifying institution, the label may serve only as a superficial identification cue. Moreover, when consumers lack trust in the source of certification or are confronted with excessive packaging information, labels may create comprehension difficulties and information overload (Rupprecht et al., 2020; Bogliacino et al., 2023; Thøgersen, 2023). This limitation is particularly relevant in the context of ICH food, because an ICH label alone may be insufficient to help consumers understand the cultural value embodied in the product. Whether the positive effects identified in prior research on certification labels can be extended to ICH labels therefore requires further examination. Based on their cultural identification and certification functions, this study proposes the following hypothesis:

*H2*: The presence of an ICH label on ICH food packaging will increase consumers’ purchase intention.

#### 2.2.3 The joint effects of storytelling and ICH labels

In actual purchase environments, consumers are typically exposed to multiple packaging cues simultaneously. Research on multiple cues suggests that when different cues support a consistent product judgment and provide information that is not entirely redundant, they may reinforce one another. By contrast, when cues convey conflicting meanings, differ in credibility, or provide highly repetitive information, the addition of another cue may fail to generate an incremental effect and may even create cognitive conflict or information redundancy (Miyazaki et al., 2005). Accordingly, the joint effects of storytelling and ICH labels should not be assumed to be inherently complementary but should instead be evaluated in light of their informational content and consistency.

In the present study, storytelling provides concrete information about a product’s historical origins, relevant individuals, and process of intergenerational transmission, whereas an ICH label offers a concise indication of the product’s ICH status. When the two cues are presented together, consumers receive both the substantive information needed to understand the product’s cultural value and a succinct basis for identifying its cultural affiliation. Research in food contexts has shown that a label alone cannot fully substitute for the specific cultural meanings conveyed through storytelling (Barretta et al., 2025). When both cues refer to the same cultural origin, the consistency between substantive cultural content and identity information may enhance consumers’ overall evaluations of the product’s cultural value and purchase suitability, thereby increasing purchase intention. Accordingly, this study proposes:

*H3*: Storytelling and ICH labels will have a positive interaction effect on purchase intention, such that the incremental effect of an ICH label on purchase intention will be stronger in the storytelling condition than in the non-storytelling condition.

### 2.3 The influence of purchase motivation on the effects of packaging cues: a construal level theory perspective

Consumers’ use of packaging cues also depends on whether those cues align with their current purchase goals. Self-purchase and gift-giving differ not only in the intended recipient but also in the considerations consumers emphasize when evaluating a product. Self-purchase primarily centers on the consumer’s own consumption experience, whereas gift-giving requires simultaneous consideration of the recipient’s likely response, the appropriateness of the gift, and the product’s ability to convey a particular meaning. Consequently, the same packaging cue may be assigned different diagnostic weights under the two purchase motivations.

Construal level theory provides a theoretical explanation for this difference. Trope and Liberman proposed that psychological distance comprises temporal, spatial, social, and hypothetical distance. When psychological distance is relatively short, individuals tend to adopt concrete and contextualized low-level construals, focusing on how an action can be performed and whether it is feasible. When psychological distance is greater, individuals are more likely to adopt abstract and generalized high-level construals, emphasizing the action’s central purpose, value, and desirability (Trope and Liberman, 2010). The distinction between the self and others represents an important form of social distance. Compared with making decisions for oneself, making decisions for others typically requires individuals to move beyond their immediate personal experiences and evaluate the product from the perspective of the recipient and the relational context. Such decisions are therefore more likely to increase attention to the product’s overall meaning and core value (Lu et al., 2013).

This reasoning is further supported by research on gift-giving. Consumers purchasing for themselves tend to focus more directly on whether a product matches their personal preferences, how it will be used, and the experience they expect it to provide. Gift-givers, by contrast, place greater emphasis on whether a gift carries symbolic meaning, communicates their intentions, and is likely to receive social approval (Lyu et al., 2022; Baskin et al., 2014). Purchase motivation may therefore alter the relative importance consumers attach to concrete experiential information and abstract cultural information.

Storytelling provides concrete and imaginable cultural content and may therefore possess greater diagnostic value in the self-purchase context. An ICH label, by contrast, signals cultural affiliation through a concise and abstract symbol and may consequently receive greater attention in the gift-giving context. Because gift-giving involves both the communication of cultural meaning and the expression of identity, the combination of storytelling and an ICH label may more closely correspond to gift-givers’ evaluative needs. Accordingly, this study proposes the following hypotheses:

*H4*: Purchase motivation moderates the effect of storytelling on purchase intention. Specifically, the positive effect of storytelling will be stronger in the self-purchase context than in the gift-giving context.

*H5*: Purchase motivation moderates the effect of an ICH label on purchase intention. Specifically, the positive effect of an ICH label will be stronger in the gift-giving context than in the self-purchase context.

*H6*: Storytelling, ICH labels, and purchase motivation will exhibit a three-way interaction effect. Specifically, the positive interaction between storytelling and ICH labels will be stronger in the gift-giving context than in the self-purchase context.

### 2.4 The mediating and conditional indirect effects of cultural identity

Cultural identity generally refers to an individual’s understanding, acceptance, sense of belonging, and positive affect toward a particular culture (He and Wang, 2015; Clark, 1990). Prior research has predominantly conceptualized cultural identity as a relatively stable identity orientation and examined how global–local identity or national cultural identity influences preferences for local foods, domestic brands, and imported brands (He and Wang, 2015; Zhang et al., 2022). Related studies have also identified cultural identity as an important antecedent of purchase intention for cultural products (Dong and Li, 2025).

Recent research has begun to examine the effects of cultural information conveyed through packaging. For example, traditional cultural embeddedness may influence purchase intention for tourism souvenirs through cultural identity and perceived value (Huang et al., 2025), while textual and non-textual presentations of ICH elements may shape consumers’ brand preferences through perceived value (Wang et al., 2025). However, these studies have mainly focused on consumers’ general cultural identity, the overall intensity of cultural information on packaging, or the presentation formats of heritage elements. They have not distinguished between storytelling and ICH labels as two specific packaging cues, nor have they adequately explained how these cues relate to consumers’ cultural understanding of, emotional connection with, and identification with a particular ICH food.

Accordingly, this study introduces cultural identity with a specific ICH food to explain the potential psychological process through which different cultural cues on packaging influence purchase intention. In the present study, cultural identity specifically refers to consumers’ cognitive recognition of, positive affect toward, and tendency to identify with the local ICH food culture embodied in Caishiji dried tofu. Rather than treating cultural identity as a general identity orientation, this study examines its role within the specific context of ICH food and packaging communication, thereby providing further insight into the relationship between external cultural cues and consumers’ purchase responses.

Narrative processing can help consumers understand the meaning of a product and establish connections with a brand (Edson Escalas, 2004), whereas an ICH label can signal the association between a product and a specific cultural tradition (Huang et al., 2025). Building on the preceding analysis of these two cues, this study conceptualizes cultural identity as a potential psychological mechanism through which cultural cues on packaging influence purchase intention. Accordingly, the following hypotheses are proposed:

*H7*: Cultural identity mediates the effects of packaging cues on purchase intention.

*H7a*: Storytelling indirectly increases consumers’ purchase intention by enhancing cultural identity.

*H7b*: An ICH label indirectly increases consumers’ purchase intention by enhancing cultural identity.

*H7c*: The interaction between storytelling and an ICH label indirectly increases consumers’ purchase intention by enhancing cultural identity.

Purchase motivation may also shape the process through which packaging cues foster cultural identity. As suggested by the preceding analysis based on construal level theory, self-purchase places greater emphasis on concrete consumption experiences, whereas gift-giving directs greater attention to cultural meaning and identity expression (Lyu et al., 2022; Lu et al., 2013). Accordingly, this study expects the indirect effect of storytelling to be stronger in the self-purchase context, whereas the indirect effect arising from the combination of storytelling and an ICH label is expected to be stronger in the gift-giving context. Because the existing literature provides insufficient grounds for specifying the direction of the indirect effect of an ICH label alone, this study predicts only that this indirect effect will be moderated by purchase motivation. Accordingly, the following hypotheses are proposed:

*H8*: Purchase motivation moderates the indirect effects of packaging cues on purchase intention through cultural identity.

*H8a*: The indirect effect of storytelling on purchase intention through cultural identity will be stronger in the self-purchase context than in the gift-giving context.

*H8b*: Purchase motivation moderates the indirect effect of an ICH label on purchase intention through cultural identity.

*H8c*: The indirect effect of the interaction between storytelling and an ICH label on purchase intention through cultural identity will be stronger in the gift-giving context than in the self-purchase context.

### 2.5 Theoretical framework

This study adopts cue utilization theory as its overarching theoretical framework. The theory emphasizes that consumers select and integrate different cues according to their informational value, credibility, and relevance to the current decision goal. Accordingly, this study conceptualizes storytelling and ICH labels as two qualitatively distinct cultural cues on packaging and examines their independent and combined effects to explain how multiple cultural cues on ICH food packaging jointly shape consumer judgments.

Within this cue utilization process, cultural identity is introduced to explain the psychological link between cultural information conveyed on packaging and purchase intention. Consumers’ cultural understanding of, recognition of, and emotional connection with a specific ICH food may constitute an important mechanism through which external cultural cues influence purchase responses. In addition, drawing on construal level theory, this study incorporates purchase motivation into the cue utilization process as a contextual boundary condition and examines whether it further alters the indirect effects of packaging cues on purchase intention through cultural identity.

On this basis, the study develops a conditional process framework centered on cue utilization theory that integrates cue integration, psychological mechanisms, and contextual boundary conditions. To test the proposed relationships, the study employs a 2 (storytelling: absent vs. present) × 2 (ICH label: absent vs. present) × 2 (purchase motivation: self-purchase vs. gift-giving) between-subjects experimental design. The proposed theoretical model is presented in Figure 1.

**Figure 1:**
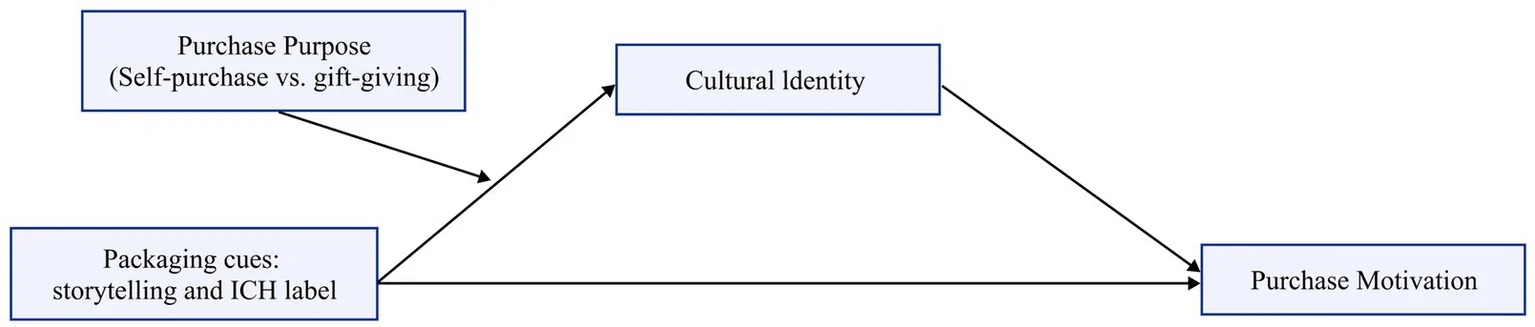
Research framework diagram.

## 3 Methods

### 3.1 Research design and stimulus materials

This study employed a 2 (storytelling: absent vs. present) × 2 (ICH label: absent vs. present) × 2 (purchase motivation: self-purchase vs. gift-giving) between-subjects experimental design. The combinations of storytelling and the ICH label produced four packaging stimuli, each of which was presented in both the self-purchase and gift-giving contexts, resulting in eight experimental conditions. Each participant was randomly assigned to one condition and was exposed to only one purchase context and its corresponding packaging stimulus.

Caishiji dried tofu was selected as the focal product for two main reasons. First, it possesses the characteristics of both a traditional local food and an ICH food with distinctive cultural connotations. Second, the product has relatively limited nationwide recognition, which helps minimize potential interference from participants’ pre-existing brand impressions or consumption experience.

For the storytelling manipulation, drawing on prior research on storytelling text used in food packaging, the narrative incorporated information concerning the product’s origins, traditional production techniques, and raw ingredients. The text was kept brief to ensure readability and approximate an authentic packaging context (Barretta et al., 2025; Fenger et al., 2015; Jaud et al., 2023). The storytelling text was as follows: “According to local legend, during the Qing Dynasty, an elderly couple operated a small teahouse at the foot of Cuiluo Mountain in Caishiji. Seeking to create a snack better suited to accompany tea, they experimented repeatedly and eventually braised dried tofu with soybeans, licorice, and other ingredients. The resulting product had a reddish-brown color resembling that of brewed tea. Tea drinkers found that its savory flavor and lingering sweetness paired particularly well with tea and consequently named it ‘tea dried tofu.’ Upon hearing of the delicacy, the Jiaqing Emperor ordered that it be presented to the imperial court. Caishiji dried tofu subsequently became a tribute food from the Jiangnan region and has been passed down to the present day.”

To minimize potential visual bias arising from differences in the amount of textual information across conditions, the non-storytelling condition included a basic product description that contained no narrative cultural content. This design ensured that differences between conditions were attributable primarily to the storytelling manipulation itself. The conventional product description was as follows: “Caishiji dried tofu is made primarily from soybeans and has the mild aroma and firm texture commonly associated with soybean-based foods. The product has a relatively dark appearance and a subtle lingering sweetness after chewing. It can be consumed directly as an everyday snack, paired with tea or porridge, or used in home cooking. Its overall flavor is mild, and it can be consumed in a variety of ways, reflecting the everyday consumption characteristics of traditional soybean-based foods.”

Both texts included information about the product’s ingredients, color and flavor, tea-pairing or consumption contexts, and traditional food attributes. Compared with the conventional product description, the storytelling text additionally incorporated a narrative structure comprising temporal setting, characters, event development, and outcomes. Excluding punctuation marks, the conventional product description and storytelling text contained approximately 104 and 130 Chinese characters, respectively, and occupied six and seven lines on the packaging. Both texts used the same typeface, font size, text color, and fixed text area and were positioned beneath the product name.

The ICH label manipulation was implemented by adding or removing the same official ICH symbol at a fixed position in the upper-left corner of the packaging. Apart from the textual content and the presence or absence of the ICH label, the four packaging stimuli were identical in terms of bag format, product name, background image, net content, layout structure, and display size. The complete stimulus materials are presented in Figure 2.

**Figure 2:**
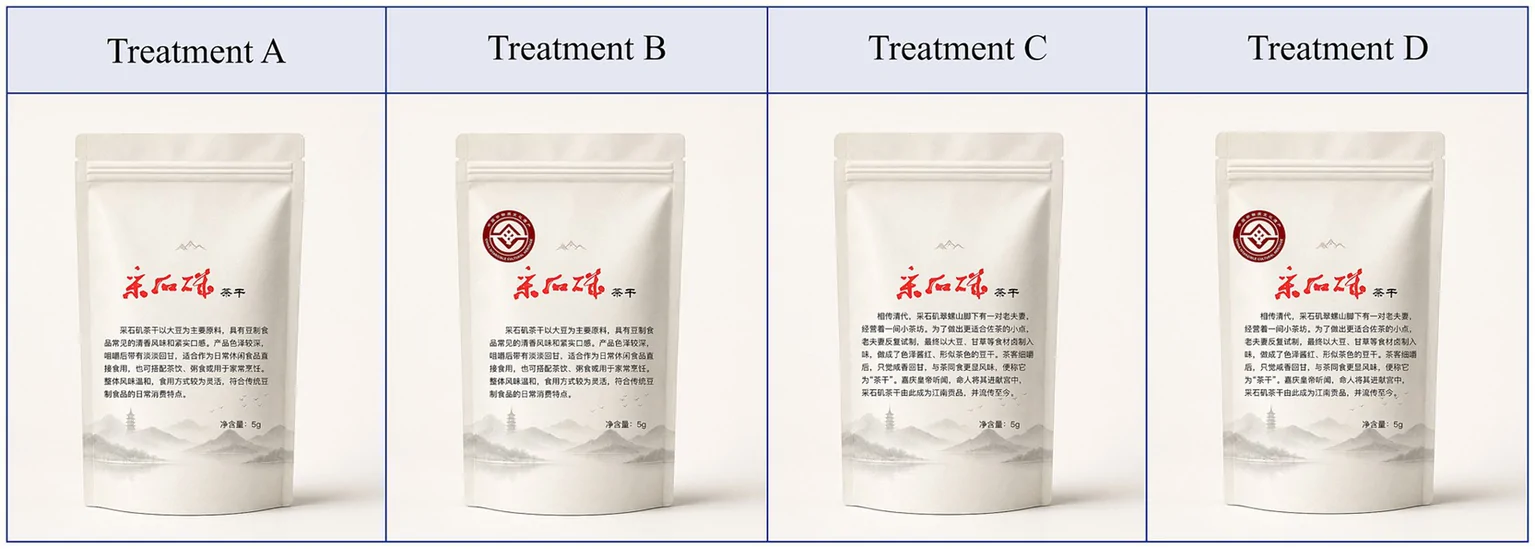
Experimental materials.

### 3.2 Experimental contexts and procedure

#### 3.2.1 Scenario manipulation of purchase motivation

Upon entering the questionnaire, participants were first randomly assigned to either the self-purchase context or the gift-giving context. Because the identity of the gift recipient, the closeness of the relationship, the social importance of the gift-giving occasion, and the perceived risk associated with gift selection may influence consumers’ evaluations of packaging, these factors were standardized in the gift-giving context.

In the self-purchase context, participants read the following scenario: “Please imagine that you are traveling away from home and pass by a store selling local traditional foods. You plan to purchase some local specialties for your own consumption after the trip. The product will not be used for entertaining guests or given to anyone else. You have a sufficient purchasing budget, and your decision regarding whether to purchase the product will depend primarily on your overall impression of its packaging and the information presented on it.”

In the gift-giving context, participants read the following scenario: “Please imagine that you are traveling away from home and pass by a store selling local traditional foods. You plan to purchase some local specialties for a close friend of approximately the same age as yourself, to be given as an ordinary travel souvenir after the trip. The gift-giving occasion does not involve a birthday, wedding, major festival, business interaction, or any other formal occasion, and the recipient has no known relevant dietary restrictions. You have a sufficient purchasing budget, and your decision regarding whether to purchase the product will depend primarily on your overall impression of its packaging and the information presented on it.”

The gift recipient was specified as a close friend of approximately the same age as the participant to minimize potential confounding effects arising from generational differences, family roles, and social-status relationships. The gift-giving occasion was defined as the presentation of an ordinary travel souvenir, while formal gift-giving occasions and known dietary restrictions were excluded. These controls helped reduce variation across gift-giving situations in terms of social importance and the perceived risk of gift–recipient mismatch. Except for the intended recipient and the purchase-motivation manipulation, the two contexts were identical with respect to the travel setting, retail environment, product category, budget conditions, and basis for the purchase decision.

#### 3.2.2 Presentation of packaging stimuli and questionnaire procedure

After reading the purchase scenario, participants were randomly assigned to one of four packaging stimulus conditions: a conventional product description without an ICH label, a conventional product description with an ICH label, storytelling without an ICH label, or storytelling with an ICH label. The combination of purchase motivation and packaging stimuli resulted in eight experimental conditions. Each participant was exposed to only one purchase context and one packaging stimulus.

While the packaging stimulus was displayed, the questionnaire system automatically recorded the amount of time each participant spent on the packaging page. To prevent the manipulation-check items from revealing the purpose of the study or further directing participants’ attention toward storytelling, the ICH label, or the intended recipient of the purchase, participants first completed the measures of the focal variables based on the purchase scenario and packaging stimulus they had viewed. These measures included the cultural identity scale and the purchase intention scale. Participants subsequently completed manipulation checks for storytelling, the ICH label, and purchase motivation, followed by measures of potential confounding factors, including the perceived credibility and attractiveness of the packaging information, perceived amount of information, text readability, and visual salience. Finally, participants responded to items assessing prior knowledge, cultural familiarity, previous product consumption experience, and demographic characteristics.

All participants completed the questionnaire in the same sequence of pages. The questionnaire system also recorded each participant’s total completion time, which was used as one of the criteria for subsequent data-quality screening.

#### 3.2.3 Measures for the manipulation checks

With the exception of the label-recognition item, all manipulation-check items were measured using a 7-point Likert scale ranging from 1 (“strongly disagree”) to 7 (“strongly agree”). The complete set of items is provided in Supplementary material S1.

To assess the manipulation of purchase motivation, this study drew on the direct measures of self-focus and other-focus developed by Weisfeld-Spolter et al. (2015) and adapted them to the present research context. The manipulation check comprised two items: “I am purchasing this product primarily for my own consumption” and “I am purchasing this product primarily as a gift for someone else.” These items were used to determine whether participants accurately perceived the intended self-purchase and gift-giving motivations, respectively (Weisfeld-Spolter et al., 2015).

To assess the storytelling manipulation, the storytelling condition was compared with the conventional product-description condition. Three items measuring perceived narrativity were developed contextually based on Woodside, 2010 characterization of stories in terms of characters, events, and temporal development, together with the manipulation approach used by Fenger et al. (2015) in their research on brief stories presented on food packaging. These items were designed to verify that participants perceived the storytelling text as genuinely narrative rather than merely as containing a greater amount of information (Woodside, 2010; Fenger et al., 2015).

To rule out differences between the storytelling and conventional product-description conditions in other informational attributes, the study also measured packaging-information credibility, packaging attractiveness, perceived amount of information, text readability, visual salience, perceived emotionality, message valence, and perceived cognitive effort. The packaging-information credibility items were adapted from (Appelman and Sundar, 2016) message credibility scale to examine whether the two text conditions differed in perceived truthfulness and credibility. The packaging-attractiveness items were based on Lavie and Tractinsky (2004) perceived visual aesthetics scale and were simplified and adapted to the food-packaging context.

The two items measuring perceived emotionality were informed by Puntoni et al. (2009) approach to assessing the perceived emotional intensity of marketing communications. They examined, respectively, the emotional tone of the packaging text and the extent to which it elicited an emotional response (Puntoni et al., 2009). The two message-valence items were developed based on Bolls et al., 2001 distinction between positive and negative advertising valence and Briesemeister et al., 2012 measurement of informational positivity and negativity. These items were used to determine whether the two texts differed in the degree of positivity conveyed in their product information (Bolls et al., 2001; Briesemeister et al., 2012). The two perceived-cognitive-effort items were adapted to the packaging-text reading context from Paas (1992) subjective mental-effort measure and the marketing-information processing-fluency scale developed by Kostyk et al. (2021). They assessed the subjective cognitive effort participants invested in understanding and processing the packaging text (Paas, 1992; Kostyk et al., 2021). All of these additional items were measured using 7-point Likert scales.

The items measuring perceived amount of information, text readability, and visual salience were developed according to the conceptual meanings of the respective constructs and adapted to the stimulus materials used in this study. They were included to rule out alternative explanations arising from differences between the two text conditions in perceived informational richness, clarity of textual presentation, and attention-capturing capacity. The questionnaire system also recorded the amount of time participants spent viewing the packaging page to compare the objective processing duration of the two texts. It should be emphasized that viewing time was used solely as an indicator of information-processing duration and was not intended to replace participants’ subjective reports of cognitive effort.

For the ICH label manipulation, the study included items assessing label recognition and understanding of official certification. The label-recognition item examined whether participants noticed the ICH symbol on the packaging, whereas the official-certification-understanding item examined whether participants interpreted the symbol as representing formal or official ICH certification.

### 3.3 Measures

This study employed established scales to measure the key variables, with item wording appropriately adapted to the context of Caishiji dried tofu.

In this study, cultural identity with the focal ICH food refers to the extent to which consumers recognize, value, and identify with the local food culture embodied in Caishiji dried tofu. Hereafter, this construct is referred to as cultural identity. The cultural identity scale was primarily adapted from the scale developed by Fu and Luo (2023) for a specific cultural heritage context. Their scale was developed on the basis of prior research on cultural identity by Phinney, Lee and Yoo, Wang and Chen, and Ferguson and Burkhalter, and systematically validated the cognitive, affective, and behavioral dimensions of cultural identity (Phinney, 1992; Lee and Yoo, 2004; Ferguson and Burkhalter, 2015; Fu and Luo, 2023).

To align the scale with the context of ICH food packaging evaluation, the original scale was shortened and contextually adapted. Items concerning the historical age and geographical location of cultural heritage, knowledge of cultural artifacts, participation in cultural tourism activities, and the purchase of tourism-related cultural products were excluded because they did not fully correspond to the present research context. By contrast, items relating to cultural understanding, recognition of cultural value, positive affect, willingness to learn more, and cultural sharing were retained and reworded to reflect the context of Caishiji dried tofu.

The purchase intention scale was developed with reference to Bambauer-Sachse and Grewal, 2011, Ar and Kara (2012), Vilnai-Yavetz and Koren, 2013, and Huang et al., 2025. It assessed purchase intention in terms of participants’ current inclination to purchase, likelihood of purchasing in the future, and willingness to recommend the product (Huang et al., 2025; Bambauer-Sachse and Grewal, 2011; Ar and Kara, 2012; Vilnai-Yavetz and Koren, 2013).

All scale items were originally drawn from English-language sources. The research team first translated the items into Chinese and adapted them to the context of Caishiji dried tofu and ICH food packaging. The resulting Chinese questionnaire was used in both the pretest and the formal survey. During manuscript preparation, the Chinese items administered to participants were back-translated into English and cross-checked against the original scales to ensure consistency in construct meaning and semantic expression across the Chinese and English versions. All items were measured using a 7-point Likert scale ranging from 1 (“strongly disagree”) to 7 (“strongly agree”), with higher scores indicating stronger agreement with the corresponding statements. The specific measurement items are presented in Table 1.

**Table 1:** Measurement variables and items.

Variable	Measurement item
Cultural identity	I understand the local food culture embodied in Caishiji dried tofu.
	I believe that Caishiji dried tofu represents a distinctive local traditional culture.
	I believe that this traditional food embodies cultural values worthy of preservation and transmission.
	I feel a sense of familiarity or closeness when I see this traditional food.
	I have positive feelings toward and identify with the culture represented by Caishiji dried tofu.
	I am willing to learn more about the ICH food story behind Caishiji dried tofu.
	I am willing to introduce or share the cultural meaning of this traditional food with others.
Purchase intention	I would like to purchase this dried tofu product.
	I would be likely to purchase this dried tofu product in the future.
	I would recommend this dried tofu product to my friends.

Before the formal survey, a small-sample pretest was conducted, yielding 45 valid responses. The sample comprised 23 women (51.1%) and 22 men (48.9%), with a mean age of 32.00 years (SD = 7.88). Participants were drawn from East China, Central China, South China, North China, Southwest China, and Northwest China, and 64.4% held at least a bachelor’s degree. In addition, 62.2% of participants reported either no knowledge of Ma’anshan culture or only having heard of it. The pretest sample thus exhibited a reasonable degree of diversity in gender, age, educational attainment, and regional background, making it suitable for assessing item comprehensibility and conducting a preliminary evaluation of the measurement structure.

The pretest results showed that the overall Kaiser–Meyer–Olkin value was 0.838, and Bartlett’s test of sphericity was significant, *χ*^2^(45) = 244.44, *p* < 0.001, indicating that the inter-item correlation structure was suitable for factor analysis. When cultural identity and purchase intention were specified as two correlated latent variables, confirmatory factor analysis indicated that the two-factor measurement model demonstrated a good fit to the data, *χ*^2^(34) = 38.88,*p* = 0.259,*χ*^2^/df = 1.14, CFI = 0.978, TLI = 0.971, RMSEA = 0.057, SRMR = 0.072.

The standardized factor loadings of the seven cultural identity items ranged from 0.657 to 0.824, whereas those of the three purchase intention items were 0.816, 0.857, and 0.784, respectively. The recommendation-intention item had a factor loading of 0.784, indicating that it could jointly reflect purchase intention together with current purchase inclination and future purchase likelihood. For the cultural identity scale, Cronbach’s *α* was 0.900, composite reliability was 0.901, and average variance extracted was 0.567. For the purchase intention scale, Cronbach’s *α* was 0.860, composite reliability was 0.860, and average variance extracted was 0.672. The correlation between cultural identity and purchase intention was 0.547, which was lower than the square roots of the AVE values for the two constructs, 0.753 and 0.820, respectively. The HTMT value was 0.533, below the threshold of 0.85. These results indicate that the two constructs exhibited satisfactory internal consistency, convergent validity, and discriminant validity. Accordingly, all items were retained for the formal survey.

The formal survey results provided further support for the stability of the scales. The standardized factor loadings of the seven cultural identity items ranged from 0.728 to 0.784, with Cronbach’s *α* = 0.901, composite reliability = 0.902, and AVE = 0.567. The standardized factor loadings of the three purchase intention items ranged from 0.812 to 0.827, with Cronbach’s *α* = 0.862, composite reliability = 0.862, and AVE = 0.675. The correlation between the two constructs was 0.579, which was lower than their respective square roots of AVE, 0.753 and 0.822. The HTMT value was 0.657, providing further evidence of convergent and discriminant validity. Subsequently, the mean scores of the seven cultural identity items and the three purchase intention items were calculated separately and used as the overall scores for the corresponding variables.

### 3.4 Sampling and data collection

This study targeted consumers who had recently purchased local ICH food. Following established practices in related research, non-probability sampling was adopted to improve access to the target population. Participants were recruited through social media and the researchers’ interpersonal networks using convenience sampling, supplemented by snowball sampling in which respondents were invited to share the questionnaire with others. It should be noted that individuals who voluntarily chose to participate in and complete the online questionnaire may have been more attentive to or familiar with local ICH food than consumers in general. In addition, questionnaire distribution through social networks may have increased the likelihood that individuals with similar interests and backgrounds entered the sample. The study may therefore be subject to self-selection bias, and the sample is not probabilistically representative. Caution is consequently required when generalizing the findings to the broader consumer population.

To ensure adequate statistical power for detecting the focal interaction effects, an *a priori* sample-size analysis was conducted using G*Power 3.1. The test family was specified as F tests, the statistical test as “ANOVA: Fixed effects, special, main effects and interactions,” and the type of power analysis as “*A priori*.” The study employed a 2 (storytelling: absent vs. present) × 2 (ICH label: absent vs. present) × 2 (purchase motivation: self-purchase vs. gift-giving) between-subjects experimental design comprising eight conditions. Because the study focused on the joint effects of storytelling, the ICH label, and purchase motivation, the sample-size calculation was based on the theoretically central and comparatively difficult-to-detect three-way interaction, with one numerator degree of freedom. Given that interaction effects are generally smaller than main effects and that prior research provides no directly comparable estimate of the three-way interaction examined here, a relatively conservative small-to-medium effect size of *f* = 0.20 was assumed. The significance level was set at *α* = 0.05 and statistical power at 1 − *β* = 0.95. The analysis indicated that a minimum total sample of 327 participants was required to detect the three-way interaction. The final valid sample of 428 exceeded this minimum requirement.

Data were collected through an online questionnaire. To ensure the relevance of the sample to the research context, an initial screening criterion was applied so that only prospective consumers who had recently purchased local ICH food were eligible to participate. In addition, because consumers residing in Ma’anshan might respond differently to Caishiji dried tofu and its cultural information owing to greater familiarity with the local culture, recruitment primarily targeted consumers living outside Ma’anshan to reduce potential interference from local cultural familiarity. Before entering the formal experiment, respondents provided basic background information, including gender, age, and educational attainment. Their prior product knowledge, defined as their familiarity with Caishiji dried tofu, was also assessed in advance. Prior knowledge was measured using three items: “Before participating in this survey, I was relatively familiar with Caishiji dried tofu,” “Before participating in this survey, I understood the main product characteristics of Caishiji dried tofu,” and “Before participating in this survey, I was familiar with the place of origin and the related cultural background of Caishiji dried tofu.” All items were rated on a 5-point Likert scale ranging from 1 (“strongly disagree”) to 5 (“strongly agree”), and the mean of the three items was used as the participant’s prior product knowledge score.

A total of 456 questionnaires were collected. The questionnaire platform used a random-assignment procedure with equal initial quotas to allocate participants across the eight conditions in the 2 (storytelling: absent vs. present) × 2 (ICH label: absent vs. present) × 2 (purchase motivation: self-purchase vs. gift-giving) between-subjects design, with 57 participants initially assigned to each condition. Upon entering the questionnaire, participants were randomly presented with one experimental condition by the system, and each participant was permitted to complete the questionnaire only once.

The data-exclusion criteria were established before examining the outcome variables, including cultural identity and purchase intention. Considering the length of the questionnaire, including the scenario materials, packaging stimulus, and measurement items, 90 s was specified as the minimum reasonable time required to complete the questionnaire. First, 24 participants who completed the questionnaire in less than 90 s were excluded. Among the remaining 432 participants, three pre-specified condition-identification items were then used to determine whether they had correctly identified the storytelling condition, the presence or absence of the ICH label, and the intended recipient of the purchase. Participants who incorrectly identified any experimental condition to which they had actually been exposed were classified as having failed the condition-identification check. Four participants were excluded on this basis. The final sample therefore comprised 428 valid questionnaires, corresponding to a valid response rate of 93.8%.

The eight experimental conditions ultimately contained 54, 54, 54, 54, 53, 53, 53, and 53 participants, respectively, indicating that group sizes remained highly balanced. Exclusion rates did not differ significantly across the eight conditions, *χ*^2^(7) = 0.61, *p* = 0.999, indicating that exclusions were not concentrated in any particular experimental condition.

Respondents’ demographic characteristics and their cultural and consumption-related backgrounds are reported in Table 2. Randomization checks revealed no significant differences across the eight experimental groups in gender, age, educational attainment, current region of residence, familiarity with Ma’anshan culture, prior consumption of Caishiji dried tofu, frequency of purchasing local food, familiarity with official ICH labels, or prior product knowledge (*ps* ≥ 0.193). These results indicate satisfactory baseline comparability across the experimental conditions following random assignment. Prior product knowledge also did not differ significantly across the eight groups, *F*(7, 420) = 0.08, *p* = 0.999, supporting the independence of the covariate from the experimental conditions.

**Table 2:** Demographic profile of the respondents (*N* = 428).

Variable	Category	Frequency (*N*)	Percentage (%)
Gender	Male	198	46.3
	Female	230	53.7
Age	18–25 years	185	43.2
	26–35 years	181	42.3
	36–45 years	62	14.5
Education	High school or below	50	11.7
	Bachelor’s degree	268	62.6
	Master’s degree or above	110	25.7
Current region of residence	Other cities in Anhui Province	96	22.4
	Other provinces or cities in the Yangtze River Delta	124	29.0
	Other areas in Central China	92	21.5
	Other regions	116	27.1
Familiarity with Ma’anshan culture	Not at all familiar	218	50.9
	Have heard of it but am unfamiliar with it	169	39.5
	Somewhat familiar	35	8.2
	Quite or very familiar	6	1.4
Prior consumption of Caishiji dried tofu	Never consumed it	377	88.1
	Consumed it once or twice	42	9.8
	Consumed it occasionally	9	2.1
Frequency of purchasing local food	High (at least once a week)	58	13.6
	Medium (1–3 times a month)	212	49.5
	Low (less than once a month)	158	36.9
Familiarity with official ICH labels	Completely unfamiliar and have never seen one	171	40.0
	Have seen one but do not understand its meaning	149	34.8
	Slightly familiar	85	19.9
	Quite or very familiar	23	5.4
Prior product knowledge (1–5 scale)	M (SD)	1.47(0.68)	—

### 3.5 Data analysis method

This study first assessed the internal consistency, convergent validity, and discriminant validity of the cultural identity and purchase intention scales, and evaluated the measurement model using confirmatory factor analysis. The effectiveness of the storytelling, ICH label, and purchase motivation manipulations was assessed using independent-samples t tests or Pearson’s chi-square tests, as appropriate.

The core hypotheses were tested using a three-way analysis of covariance (ANCOVA) with a 2 (storytelling: absent vs. present) × 2 (ICH label: absent vs. present) × 2 (purchase motivation: self-purchase vs. gift-giving) between-subjects design. Cultural identity and purchase intention were analyzed separately as dependent variables. Prior product knowledge was measured before exposure to the experimental stimuli and was prespecified as the primary covariate. Before conducting the ANCOVA, the independence of the covariate from the experimental conditions, linearity between the covariate and dependent variables, homogeneity of regression slopes, normality of residuals, homogeneity of variance, and influential observations were examined. For significant interaction effects, the raw means, standard deviations, adjusted means, standard errors, and 95% confidence intervals for the eight experimental conditions were reported. The Holm procedure was used to adjust for multiple comparisons in the simple-effects analyses.

Prior product knowledge was selected as the primary covariate because it is directly related to consumers’ understanding of the focal product’s cultural background and product characteristics and is not affected by the experimental manipulations. To assess whether the core findings depended on this specific covariate specification, extended models additionally controlled for age, educational attainment, current region of residence, familiarity with Ma’anshan culture, prior consumption of Caishiji dried tofu, frequency of purchasing local food, and familiarity with official ICH labels. HC3 heteroskedasticity-consistent standard errors were also used to examine the robustness of significant interaction effects to heteroscedasticity and influential observations.

The factorial conditional process analysis retained the factorial structure of the three-factor experiment. Storytelling (S), ICH label (L), and purchase motivation (W) were each dummy-coded as 0 or 1, with non-storytelling, no ICH label, and self-purchase coded as 0, and storytelling, the presence of an ICH label, and gift-giving coded as 1. The cultural identity equation included the main effects of all three experimental factors, all two-way interaction terms, and the three-way interaction term. To avoid assuming *a priori* that purchase motivation moderates only the relationship between packaging cues and cultural identity, the purchase intention equation retained the same factorial structure and additionally included mean-centered cultural identity and its interaction with purchase motivation. The models were specified as follows:

$$\begin{array}{l} M=\beta 0+\beta 1\;\;S+\beta 2L+\beta 3W+\beta 4\mathrm{SL}+\beta 5\mathrm{SW}\\ +\beta 6\mathrm{LW}+\beta 7\mathrm{SLW}+\beta 8C+\varepsilon_{m} \end{array}$$ (1)

$$\begin{array}{l} Y=\gamma 0+\gamma 1S+\gamma 2L+\gamma 3W+\gamma 4\;\;\mathrm{SL}+\gamma 5\;\;\;\;\mathrm{SW}+\gamma 6\mathrm{LW}\\ +\gamma 7\;\;\;\;\mathrm{SLW}+b1M+b2\mathrm{MW}+\gamma 8C+\varepsilon_{y} \end{array}$$ (2)

Here, M denotes cultural identity, Y denotes purchase intention, and C denotes prior product knowledge. The conditional indirect effect was calculated as the product of the coefficient for the “packaging cue → cultural identity” path under the corresponding experimental condition and the conditional coefficient for the “cultural identity → purchase intention” path under the relevant purchase motivation. The indirect effects and differences between them were estimated using 5,000 bootstrap resamples, with resampling stratified within the eight experimental conditions. Because cultural identity and purchase intention were measured concurrently, these findings are interpreted as conditional indirect associations consistent with the proposed theoretical pathways rather than as evidence of strict temporal causality.

In addition, a parametric Monte Carlo simulation with 10,000 replications was conducted using the regression coefficients, residual variances, and actual sample sizes of the eight experimental cells from the final conditional process model as population parameters. In each replication, a sample of *N* = 428 was generated and both regression equations were re-estimated. An indirect effect was considered successfully detected when both component paths constituting the corresponding conditional indirect effect simultaneously reached statistical significance at *p* < 0.05.

## 4 Results analysis

### 4.1 Manipulation check results

The manipulation check for storytelling showed that participants in the storytelling condition reported significantly higher perceived narrativity than those in the conventional product-description condition (Mstorytelling = 5.46, SD = 0.79; Mconventional description = 3.10, SD = 0.92; *t*(415.59) = 28.51, *p* < 0.001, Cohen’s *d* = 2.76). This result indicates that participants clearly perceived the storytelling text as possessing a narrative form characterized by characters, events, and temporal or causal development, rather than merely as general product information. The storytelling manipulation was therefore successful.

To rule out alternative explanations arising from differences between the storytelling and conventional product-description conditions in other informational attributes, the study further compared perceived amount of information, text readability, visual salience, packaging attractiveness, overall information credibility, perceived emotionality, message valence, perceived cognitive effort, and packaging-viewing time across the two conditions. None of these between-group differences was statistically significant (|*t*| ≤ 0.96, *ps* ≥ 0.340, |*d*| ≤ 0.09). Thus, no systematic differences were observed between the storytelling and conventional product-description conditions in perceived informational richness, ease of reading, attention-capturing capacity, visual attractiveness, credibility, emotional tone, positivity of information, subjective processing effort, or objective viewing duration.

The manipulation check for the ICH label showed that 95.3% of participants in the ICH-label condition correctly identified the ICH symbol on the packaging, whereas no participant in the no-label condition reported identifying the symbol. The difference was statistically significant, *χ*^2^(1) = 389.79, *p* < 0.001, Cramér’s V = 0.95. Participants in the ICH-label condition also demonstrated significantly greater understanding of the product’s ICH status than those in the no-label condition (MICH label = 5.37, SD = 0.98; Mno label = 3.14, SD = 1.04; *t*(424.54) = 22.79, *p* < 0.001, *d* = 2.20). Their understanding of the product as having received formal or official ICH recognition was likewise significantly higher (MICH label = 5.22, SD = 1.01; Mno label = 2.84, SD = 0.93; *t*(422.98) = 25.29, *p* < 0.001, *d* = 2.44). These results indicate that participants not only noticed the ICH label but also understood the ICH status and official recognition conveyed by it. The ICH label manipulation was therefore successful.

The manipulation check for purchase motivation showed that participants in the self-purchase context scored significantly higher than those in the gift-giving context on the item “I am purchasing this product primarily for my own consumption” (Mself-purchase = 6.07, SD = 0.97; Mgift-giving = 1.92, SD = 0.94; *t*(425.97) = 44.89, *p* < 0.001, *d* = 4.34). Conversely, participants in the gift-giving context scored significantly higher than those in the self-purchase context on the item “I am purchasing this product primarily as a gift for someone else” (Mgift-giving = 6.09, SD = 0.94; Mself-purchase = 1.93, SD = 0.93; *t*(425.48) = 46.07, *p* < 0.001, *d* = 4.45). These findings indicate that participants clearly distinguished between the self-purchase and gift-giving contexts, confirming the effectiveness of the purchase motivation manipulation.

### 4.2 ANCOVA assumption checks and descriptive statistics

Before conducting the ANCOVA, the relevant statistical assumptions were examined. Prior product knowledge did not differ significantly across the eight experimental conditions, *F*(7, 420) = 0.08, *p* = 0.999, indicating that the covariate was essentially independent of the experimental conditions. The quadratic terms for prior product knowledge in relation to cultural identity and purchase intention were both nonsignificant, with *p* = 0.668 and *p* = 0.300, respectively, supporting the assumption of linear relationships between the covariate and the two dependent variables. Further diagnostic tests showed that the tests of homogeneity of regression slopes, Levene’s tests for homogeneity of variance, and Shapiro–Wilk tests of residual normality were all nonsignificant for both models. The maximum Cook’s D values were 0.023 for the cultural identity model and 0.030 for the purchase intention model, both substantially below 1. The maximum externally studentized residuals were 3.10 and 2.98, respectively. Overall, the data showed no serious violations of the ANCOVA assumptions and no evidence that the results were dominated by individual highly influential observations. Detailed results are presented in Table 3.

**Table 3:** Tests of ANCOVA assumptions and influential observations.

Dependent variable	Homogeneity of regression slopes	Levene’s test	Shapiro–wilk test of residuals	Maximum Cook’s *d*	Maximum externally studentized residual
Cultural identity	*F*(7,412) = 1.38, *p* = 0.214	*F*(7,420) = 0.53, *p* = 0.813	W = 0.997, *p* = 0.627	0.023	3.10
Purchase intention	*F*(7,412) = 1.28, *p* = 0.256	*F*(7,420) = 1.19, *p* = 0.309	W = 0.998, *p* = 0.830	0.030	2.98

To assess whether the results were sensitive to individual observations or heteroskedasticity, additional robustness analyses were conducted. After removing the observation with the largest Cook’s D in each of the two models, the significance patterns of the main effects and the three-way interaction remained unchanged. When the models were re-estimated using HC3 heteroskedasticity-consistent standard errors, the three-way interaction remained significant in both the cultural identity model and the purchase intention model, with *F* = 7.11, *p* = 0.008 and *F* = 5.35, *p* = 0.021, respectively.

Table 4 reports the raw means and standard deviations of cultural identity and purchase intention across the eight experimental conditions, together with the adjusted means, standard errors, and 95% confidence intervals after controlling for prior product knowledge. Because prior product knowledge was relatively balanced across conditions, the adjusted means were generally close to the corresponding raw means. The descriptive results showed that, in the self-purchase context, both cultural identity and purchase intention were higher in the storytelling conditions than in the non-storytelling conditions, whereas the additional effect associated with the ICH label was relatively limited. In the gift-giving context, cultural identity and purchase intention reached their highest levels when storytelling and the ICH label were presented together. By contrast, in the absence of storytelling, presenting the ICH label alone did not increase either outcome variable. This pattern provided preliminary support for the subsequent analyses of the three-way interaction and simple effects. Detailed results are presented in Table 4.

**Table 4:** Raw descriptive statistics and covariate-adjusted means across the eight experimental conditions.

Purchase motivation	Storytelling	ICH label	N	Cultural identity raw M (SD)	Cultural identity adjusted M (SE) [95% CI]	Purchase intention raw M (SD)	Purchase intention adjusted M (SE) [95% CI]
Self-purchase	Absent	Absent	54	4.03 (0.65)	4.03 (0.08) [3.871, 4.193]	4.09 (0.70)	4.09 (0.09) [3.915, 4.264]
Self-purchase	Absent	Present	54	3.99 (0.64)	3.99 (0.08) [3.833, 4.155]	3.92 (0.71)	3.92 (0.09) [3.745, 4.094]
Self-purchase	Present	Absent	54	4.47 (0.65)	4.47 (0.08) [4.309, 4.631]	4.52 (0.79)	4.52 (0.09) [4.347, 4.695]
Self-purchase	Present	Present	54	4.57 (0.60)	4.57 (0.08) [4.412, 4.734]	4.60 (0.64)	4.60 (0.09) [4.430, 4.778]
Gift-giving	Absent	Absent	53	3.92 (0.61)	3.92 (0.08) [3.756, 4.082]	4.02 (0.67)	4.02 (0.09) [3.846, 4.198]
Gift-giving	Absent	Present	53	3.63 (0.58)	3.62 (0.08) [3.460, 3.785]	3.66 (0.61)	3.65 (0.09) [3.478, 3.830]
Gift-giving	Present	Absent	53	4.87 (0.54)	4.87 (0.08) [4.704, 5.030]	4.94 (0.53)	4.95 (0.09) [4.770, 5.121]
Gift-giving	Present	Present	53	5.34 (0.57)	5.34 (0.08) [5.175, 5.500]	5.42 (0.58)	5.42 (0.09) [5.243, 5.595]

Adjusted means were estimated at the overall mean of prior product knowledge.

### 4.3 Effects of packaging information cues and purchase motivation on cultural identity

After controlling for prior product knowledge, storytelling had a significant main effect on cultural identity, *F*(1, 419) = 249.83, *p* < 0.001, ηp^2^ = 0.374. The main effect of purchase motivation was also significant, *F*(1, 419) = 8.44, *p* = 0.004, ηp^2^ = 0.020, whereas the average independent effect of the ICH label was not significant, *F*(1, 419) = 1.06, *p* = 0.305, ηp^2^ = 0.003. The storytelling × ICH label interaction, storytelling × purchase motivation interaction, and storytelling × ICH label × purchase motivation interaction were all significant, whereas the ICH label × purchase motivation interaction was not significant. The complete results are reported in Table 5.

**Table 5:** Three-way ANCOVA for cultural identity.

Variable	*F*	*p*	Effect size (*ηp*^2^)
S	249.83	< 0.001	0.374
L	1.06	0.305	0.003
W	8.44	0.004	0.020
S × L	15.18	< 0.001	0.035
S × W	49.93	< 0.001	0.106
L × W	0.22	0.643	0.001
S × L × W	7.25	0.007	0.017
Prior product knowledge	9.18	0.003	0.021

S represents storytelling, L represents the ICH label, and W represents purchase motivation.

The interaction plot in Figure 3 further illustrates these patterns. In the self-purchase context, cultural identity was consistently higher under the storytelling conditions than under the non-storytelling conditions, while the addition of the ICH label produced little apparent change; accordingly, the two lines were approximately parallel. By contrast, in the gift-giving context, the separation between the two label conditions was more pronounced, with the highest level of cultural identity observed when storytelling and the ICH label were presented together.

**Figure 3:**
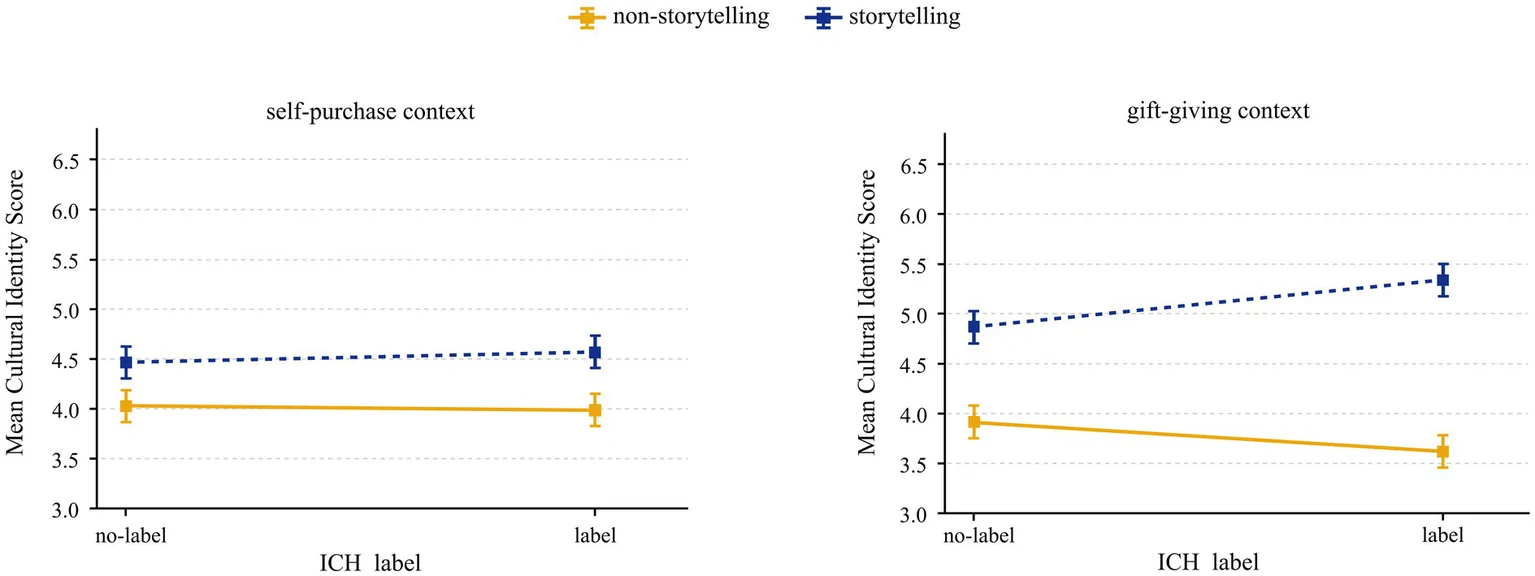
Three-way interaction effects of packaging information cues and purchase motivation on cultural identity.

Given the significant three-way interaction, simple-effects analyses were conducted, with the results reported in Table 6. Holm-adjusted simple-effects tests showed that storytelling significantly increased cultural identity across all four combinations of ICH label and purchase motivation. In the self-purchase context, the effect of the ICH label was nonsignificant under both the non-storytelling and storytelling conditions. In the gift-giving context, however, the ICH label was associated with lower cultural identity in the absence of storytelling, with an adjusted mean difference of −0.30, 95% CI [−0.53, −0.07], Holm-adjusted *p* = 0.035. By contrast, when storytelling was present, the ICH label further increased cultural identity, with an adjusted mean difference of 0.47, 95% CI [0.24, 0.70], Holm-adjusted *p* < 0.001. This pattern indicates that the effect of the ICH label depends jointly on storytelling and purchase motivation.

**Table 6:** Simple effects of the interaction on cultural identity and Holm-adjusted comparisons.

Simple effect	Purchase motivation	Fixed condition	Adjusted mean difference	95% CI	Cohen’s *d*	Holm-adjusted *p*
S	Self-purchase	L = 0	0.439	[0.211, 0.666]	0.729	< 0.001
S	Self-purchase	L = 1	0.579	[0.351, 0.807]	0.962	< 0.001
S	Gift-giving	L = 0	0.948	[0.718, 1.178]	1.575	< 0.001
S	Gift-giving	L = 1	1.715	[1.485, 1.945]	2.848	< 0.001
L	Self-purchase	S = 0	−0.037	[−0.265, 0.190]	−0.062	0.750
L	Self-purchase	S = 1	0.103	[−0.125, 0.331]	0.171	0.750
L	Gift-giving	S = 0	−0.297	[−0.527, −0.067]	−0.493	0.035
L	Gift-giving	S = 1	0.470	[0.240, 0.700]	0.781	< 0.001

S represents storytelling and L represents the ICH label; 0 = absent and 1 = present.

### 4.4 Effects of packaging information cues and purchase motivation on purchase intention

After controlling for prior product knowledge, storytelling had a significant main effect on purchase intention, *F*(1, 419) = 227.92, *p* < 0.001, *ηp*^2^ = 0.353. The main effect of purchase motivation was also significant, *F*(1, 419) = 12.93, *p* < 0.001, *ηp*^2^ = 0.030, whereas the average independent effect of the ICH label was not significant, *F*(1, 419) = 0.01, *p* = 0.943, *ηp*^2^ < 0.001. The storytelling × ICH label interaction, storytelling × purchase motivation interaction, and three-way interaction were all significant, whereas the ICH label × purchase motivation interaction was not significant. Detailed results are presented in Table 7.

**Table 7:** Three-way ANCOVA for purchase intention.

Variable	*F*	*p*	Effect size (*ηp*^2^)
S	227.92	< 0.001	0.352
L	0.01	0.943	0.000
W	12.93	< 0.001	0.030
S × L	18.86	< 0.001	0.043
S × W	38.91	< 0.001	0.085
L × W	0.58	0.448	0.001
S × L × W	5.45	0.020	0.013
Prior product knowledge	11.26	< 0.001	0.026

S represents storytelling, L represents the ICH label, and W represents purchase motivation.

The interaction plot (Figure 4) showed that, in the self-purchase context, storytelling consistently increased purchase intention, whereas the incremental effect of the ICH label was limited. In the gift-giving context, the combination of storytelling and the ICH label produced the largest increase, yielding the highest level of purchase intention among the experimental conditions examined in this study.

**Figure 4:**
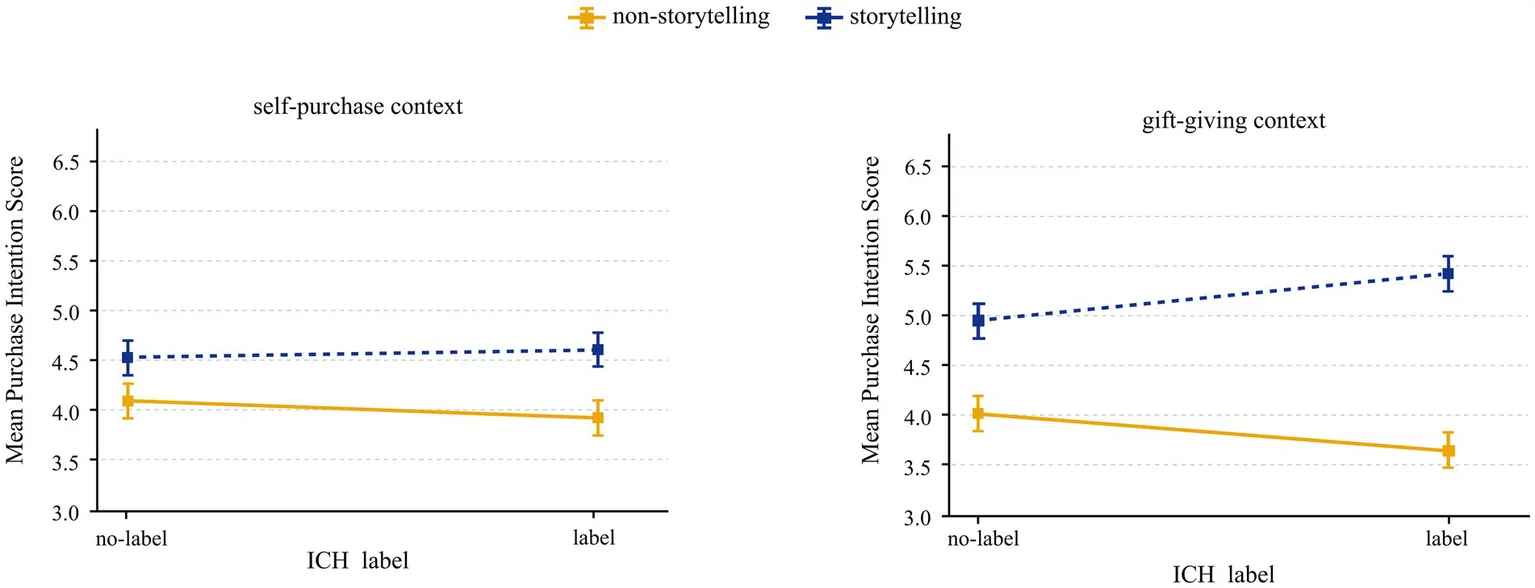
Three-way interaction effects of packaging information cues and purchase motivation on purchase intention.

Similarly, given the significant three-way interaction for purchase intention, simple interaction effects were further examined using the pooled error term from the full model, as reported in Table 8. The simple-effects results showed that storytelling significantly increased purchase intention across all four combinations of ICH label and purchase motivation. The largest storytelling effect occurred in the gift-giving context when the ICH label was present, with a mean difference of 1.765, 95% CI [1.516, 2.014], Holm-adjusted *p* < 0.001. The effect of the ICH label was nonsignificant in both conditions within the self-purchase context. In the gift-giving context, however, the ICH label decreased purchase intention when storytelling was absent but increased purchase intention when storytelling was present. These findings indicate that the ICH label did not exert a stable independent effect; rather, its influence depended on whether it was presented together with storytelling and on whether the product was the purchase motivation was self-purchase or gift-giving.

**Table 8:** Simple effects of the interaction on purchase intention and Holm-adjusted comparisons.

Simple effect	Purchase motivation	Fixed condition	Adjusted mean difference	95% CI	Cohen’s *d*	Holm-adjusted *p*
S	Self-purchase	L = 0	0.432	[0.185, 0.678]	0.662	0.003
S	Self-purchase	L = 1	0.685	[0.438, 0.931]	1.051	< 0.001
S	Gift-giving	L = 0	0.924	[0.675, 1.172]	1.417	< 0.001
S	Gift-giving	L = 1	1.765	[1.516, 2.014]	2.708	< 0.001
L	Self-purchase	S = 0	−0.170	[−0.416, 0.077]	−0.261	0.352
L	Self-purchase	S = 1	0.083	[−0.163, 0.330]	0.128	0.508
L	Gift-giving	S = 0	−0.368	[−0.617, −0.119]	−0.565	0.011
L	Gift-giving	S = 1	0.473	[0.224, 0.722]	0.726	0.001

S represents storytelling and L represents the ICH label; 0 = absent and 1 = present.

### 4.5 Factorial conditional process analysis: conditional indirect effects through cultural identity

Following the factorial conditional process model specified in Section 3.5, this study first examined the effects of packaging cues and purchase motivation on cultural identity and then assessed the effects of cultural identity, together with its interaction with purchase motivation, on purchase intention. The complete regression results are reported in Table 9.

**Table 9:** Regression results for the factorial conditional process model (*N* = 428).

Dependent variable	Predictor	B	SE	*p*	95% CI
Cultural identity	Constant	4.032	0.082	< 0.001	[3.871, 4.193]
	Prior product knowledge	0.130	0.043	0.003	[0.046, 0.215]
	Storytelling (S)	0.439	0.116	< 0.001	[0.211, 0.666]
	ICH label (L)	−0.037	0.116	0.748	[−0.265, 0.190]
	Purchase motivation (W)	−0.113	0.116	0.334	[−0.341, 0.116]
	S × L	0.140	0.164	0.393	[−0.182, 0.462]
	S × W	0.509	0.165	0.002	[0.186, 0.833]
	L × W	−0.259	0.165	0.116	[−0.583, 0.064]
	S × L × W	0.627	0.233	0.007	[0.169, 1.084]
Purchase intention	Constant	4.211	0.088	< 0.001	[4.038, 4.384]
	Prior product knowledge	0.116	0.045	0.011	[0.026, 0.205]
	Storytelling (S)	0.265	0.124	0.034	[0.020, 0.509]
	ICH label (L)	−0.156	0.121	0.199	[−0.393, 0.082]
	Purchase motivation (W)	−0.124	0.128	0.330	[−0.375, 0.126]
	S × L	0.200	0.171	0.244	[−0.137, 0.536]
	S × W	0.516	0.188	0.006	[0.146, 0.886]
	L × W	−0.167	0.173	0.336	[−0.507, 0.173]
	S × L × W	0.526	0.250	0.036	[0.035, 1.016]
	Cultural identity	0.381	0.068	< 0.001	[0.247, 0.515]
	Cultural identity × W	−0.231	0.101	0.023	[−0.430, −0.031]

S represents storytelling, L represents the ICH label, and W represents purchase motivation.

The results for the cultural identity equation were generally consistent with the significance pattern obtained from the three-way ANCOVA. Under the reference condition of self-purchase without an ICH label, storytelling significantly increased cultural identity, B = 0.439, SE = 0.116, *p* < 0.001, 95% CI [0.211, 0.666]. Under the reference condition of self-purchase without storytelling, the conditional effect of the ICH label was not significant, B = −0.037, SE = 0.116, *p* = 0.748, 95% CI [−0.265, 0.190]. The storytelling × purchase motivation interaction was significant, B = 0.509, SE = 0.165, *p* = 0.002, and the storytelling × ICH label × purchase motivation interaction was also significant, B = 0.627, SE = 0.233, *p* = 0.007. By contrast, neither the storytelling × ICH label interaction nor the ICH label × purchase motivation interaction was significant. These results indicate that the effect of storytelling on cultural identity varies as a function of purchase motivation and the ICH label configuration.

In the purchase intention equation, cultural identity was significantly and positively associated with purchase intention in the self-purchase context, B = 0.381, SE = 0.068, *p* < 0.001, 95% CI [0.247, 0.515]. The interaction between cultural identity and purchase motivation was significant and negative, B = −0.231, SE = 0.101, *p* = 0.023, 95% CI [−0.430, −0.031]. Further simple-slope analysis showed that the association between cultural identity and purchase intention remained significantly positive in the gift-giving context, although its magnitude was weaker, B = 0.150, SE = 0.076, *p* = 0.047. After controlling for cultural identity, the storytelling × purchase motivation interaction and the storytelling × ICH label × purchase motivation interaction remained significant.

The results of the 5,000-bootstrap analysis are presented in Table 10. The conditional indirect effects of storytelling were significantly positive across all four combinations of ICH label and purchase motivation, with none of the 95% bootstrap confidence intervals including zero. The conditional indirect effects of the ICH label were nonsignificant in both self-purchase conditions. In the gift-giving context without storytelling, the ICH label exhibited a negative conditional indirect effect, with an estimate of −0.045, 95% Boot CI [−0.111, −0.001]. By contrast, in the gift-giving context with storytelling, the ICH label exhibited a positive conditional indirect effect, with an estimate of 0.071, 95% Boot CI [0.006, 0.149]. These findings indicate that, in the gift-giving context, the indirect association between the ICH label and purchase intention through cultural identity depends on whether storytelling is simultaneously present.

**Table 10:** Conditional indirect effects across experimental conditions.

Indirect effect	Purchase motivation	Fixed condition	Estimate	Boot SE	95% Boot CI
S	Self-purchase	L = 0	0.167	0.056	[0.066, 0.285]
S	Self-purchase	L = 1	0.221	0.057	[0.115, 0.340]
S	Gift-giving	L = 0	0.142	0.071	[0.012, 0.290]
S	Gift-giving	L = 1	0.258	0.124	[0.020, 0.508]
L	Self-purchase	S = 0	−0.014	0.047	[−0.109, 0.079]
L	Self-purchase	S = 1	0.039	0.046	[−0.048, 0.129]
L	Gift-giving	S = 0	−0.045	0.029	[−0.111, −0.001]
L	Gift-giving	S = 1	0.071	0.037	[0.006, 0.149]

S represents storytelling and L represents the ICH label; 0 = absent and 1 = present.

The cross–purchase motivation differences in the conditional indirect effects and the indirect interaction effects are reported in Table 11. The differences in the conditional indirect effects of storytelling and the ICH label between the self-purchase and gift-giving contexts were both nonsignificant, as their bootstrap confidence intervals included zero. The indirect interaction between storytelling and the ICH label was nonsignificant in the self-purchase context, with an estimate of 0.053, 95% Boot CI [−0.077, 0.188], but was significantly positive in the gift-giving context, with an estimate of 0.115, 95% Boot CI [0.009, 0.244]. However, the difference in this indirect interaction across the two purchase motivations was not significant. The indirect three-way interaction was 0.062, 95% Boot CI [−0.113, 0.233].

**Table 11:** Differences in conditional indirect effects and indirect interactions.

Comparison type	Effect	Fixed condition or context	Estimate	Boot SE	95% Boot CI
Cross-motivation difference	S	L = 0	−0.025	0.089	[−0.196, 0.149]
	S	L = 1	0.037	0.135	[−0.225, 0.303]
	L	S = 0	−0.030	0.056	[−0.140, 0.075]
	L	S = 1	0.031	0.058	[−0.084, 0.145]
Indirect interaction	S × L	Self-purchase	0.053	0.066	[−0.077, 0.188]
	S × L	Gift-giving	0.115	0.060	[0.009, 0.244]
Indirect three-way interaction	S × L × W	Gift-giving − self-purchase	0.062	0.089	[−0.113, 0.233]

S represents storytelling, L represents the ICH label, and W represents purchase motivation; 0 = absent and 1 = present.

Overall, storytelling exhibited a significant positive conditional indirect association with purchase intention through cultural identity across different ICH label and purchase motivation conditions. In the gift-giving context, the direction of the indirect effect of the ICH label depended on whether storytelling was present. However, none of the cross–purchase motivation differences in the conditional indirect effects reached statistical significance. The current results therefore do not provide sufficient evidence that purchase motivation significantly alters the magnitude of the overall indirect effects.

The power analysis showed that, in the self-purchase context, the statistical power for the conditional indirect effects of storytelling ranged from 96.5 to 99.8%, providing adequate power to detect the storytelling-related pathways. In the gift-giving context, the power for the storytelling pathway was approximately 54.8%, primarily because the conditional association between cultural identity and purchase intention was relatively weak. Under the self-purchase condition without storytelling, the power for detecting the independent indirect effect of the ICH label was only 6.3%; therefore, the nonsignificant result for this pathway should not be interpreted as definitive evidence of the absence of an effect. In the gift-giving context, the power for the indirect effects of the ICH label ranged from 39.4 to 53.6%, and these results should likewise be interpreted with caution.

### 4.6 Robustness analyses with additional covariates and the full sample

To examine whether the main findings were sensitive to the choice of covariates, the covariate specification was first expanded using the restricted sample of 428 participants. In addition to prior product knowledge, the extended models further controlled for age, educational attainment, current region of residence, familiarity with Ma’anshan culture, prior consumption of Caishiji dried tofu, frequency of purchasing local food, and familiarity with official ICH labels. Among these variables, frequency of purchasing local food was used as a behavioral proxy for involvement with ICH food, although it cannot fully substitute for a dedicated measure of ICH involvement.

As shown in Table 12, after including these additional covariates, the direction and significance of the main experimental effects remained largely consistent with those of the original models. For cultural identity, the main effect of storytelling remained significant, *F* = 245.80, *p* < 0.001, *ηp*^2^ = 0.377; the storytelling × ICH label interaction remained significant, *F* = 13.72, *p* < 0.001, *ηp*^2^ = 0.033; the storytelling × purchase motivation interaction remained significant, *F* = 46.50, *p* < 0.001, *ηp*^2^ = 0.103; and the storytelling × ICH label × purchase motivation interaction also remained significant, *F* = 7.34, *p* = 0.007, *ηp*^2^ = 0.018. The average main effect of the ICH label remained nonsignificant, *F* = 0.84, *p* = 0.360, *ηp*^2^ = 0.002.

**Table 12:** Three-way models with extended covariates.

Effect	Cultural identity *F*	*p*	*ηp* ^2^	Purchase intention *F*	*p*	*ηp* ^2^
S	245.80	< 0.001	0.377	222.90	< 0.001	0.354
L	0.84	0.360	0.002	0.00	0.968	0.000
W	7.42	0.007	0.018	10.81	0.001	0.026
S × L	13.72	< 0.001	0.033	19.64	< 0.001	0.046
S × W	46.50	< 0.001	0.103	35.76	< 0.001	0.081
L × W	0.18	0.676	0.000	0.39	0.532	0.001
S × L × W	7.34	0.007	0.018	5.67	0.018	0.014

S represents storytelling, L represents the ICH label, and W represents purchase motivation.

The purchase intention model exhibited the same overall pattern. The main effect of storytelling was significant, *F* = 222.90, *p* < 0.001, ηp^2^ = 0.354; the storytelling × ICH label interaction was significant, *F* = 19.64, *p* < 0.001, *ηp*^2^ = 0.046; the storytelling × purchase motivation interaction was significant, *F* = 35.76, *p* < 0.001, *ηp*^2^ = 0.081; and the three-way interaction remained significant, *F* = 5.67, *p* = 0.018, *ηp*^2^ = 0.014. The average main effect of the ICH label remained nonsignificant, *F* = 0.00, *p* = 0.968, *ηp*^2^ < 0.001. These results indicate that the main experimental effects were not driven by individual differences in age, educational attainment, regional background, cultural familiarity, prior consumption experience, frequency of purchasing local food, or familiarity with ICH labels.

Building on these analyses, the three-way ANCOVA was re-estimated using the full sample of 456 questionnaires to examine whether the sample-exclusion procedure affected the main findings. As shown in Table 13, the full sample produced the same significance pattern as the restricted sample of 428 participants. For both cultural identity and purchase intention, the main effects of storytelling and purchase motivation, the storytelling × ICH label interaction, the storytelling × purchase motivation interaction, and the three-way interaction were significant in both samples, whereas the average main effect of the ICH label and the ICH label × purchase motivation interaction were nonsignificant. The partial η^2^ values were also highly similar across the two samples, with no substantive changes in either effect direction or effect magnitude. Together with the previously reported test showing no significant differences in exclusion rates across the experimental conditions, the full-sample analysis further indicates that the main experimental conclusions are not dependent on the sample-exclusion criteria.

**Table 13:** Comparison of three-way ANCOVA results between the restricted and full samples.

Dependent variable	Effect	*p* (*N* = 428)	*ηp*^2^ (*N* = 428)	*p* (*N* = 456)	*ηp*^2^ (*N* = 456)
Cultural identity	S	< 0.001	0.374	< 0.001	0.384
	L	0.305	0.003	0.207	0.004
	W	0.004	0.020	0.003	0.020
	S × L	< 0.001	0.035	< 0.001	0.036
	S × W	< 0.001	0.106	< 0.001	0.117
	L × W	0.643	0.001	0.334	0.002
	S × L × W	0.007	0.017	0.004	0.019
Purchase intention	S	< 0.001	0.352	< 0.001	0.347
	L	0.943	0.000	0.927	0.000
	W	< 0.001	0.030	< 0.001	0.025
	S × L	< 0.001	0.043	< 0.001	0.037
	S × W	< 0.001	0.085	< 0.001	0.076
	L × W	0.448	0.001	0.266	0.003
	S × L × W	0.020	0.013	0.007	0.016

S represents storytelling, L represents the ICH label, and W represents purchase motivation.

Overall, the full-sample analysis, restricted-sample analysis, and extended-covariate models yielded consistent results in terms of the direction of the core effects, significance patterns, and effect-size magnitudes. This consistency indicates that excluding participants who completed the questionnaire too quickly or failed the manipulation checks did not substantively alter the study conclusions. Based on these results, H1, H3, H6, and H7a were supported, whereas H7 and H7c received partial support. H2, H4, H5, H7b, and H8 together with all of its sub-hypotheses were not supported. It should be noted that although the moderating effect corresponding to H4 was statistically significant, the positive effect of storytelling was stronger in the gift-giving context than in the self-purchase context, which was opposite to the hypothesized direction. H7c was supported only in the gift-giving context, indicating that the indirect interaction between storytelling and the ICH label through cultural identity was context dependent. Although some conditional indirect effects exhibited different patterns across the self-purchase and gift-giving contexts, none of the corresponding cross-context differences reached statistical significance. Accordingly, H8, H8a, H8b, and H8c were not supported. A summary of the hypothesis-testing results is presented in Table 14.

**Table 14:** Hypothesis testing results.

Hypothesis	Conclusion
*H1*: Compared with a conventional product description, storytelling on ICH food packaging will increase consumers’ purchase intention.	Supported
*H2*: The presence of an ICH label on ICH food packaging will increase consumers’ purchase intention.	Not supported
*H3*: Storytelling and ICH labels will have a positive interaction effect on purchase intention, such that the incremental effect of an ICH label on purchase intention will be stronger in the storytelling condition than in the non-storytelling condition.	Supported
*H4*: Purchase motivation moderates the effect of storytelling on purchase intention. Specifically, the positive effect of storytelling will be stronger in the self-purchase context than in the gift-giving context.	Not supported (opposite direction)
*H5*: Purchase motivation moderates the effect of an ICH label on purchase intention. Specifically, the positive effect of an ICH label will be stronger in the gift-giving context than in the self-purchase context.	Not supported
*H6*: Storytelling, ICH labels, and purchase motivation will exhibit a three-way interaction effect. Specifically, the positive interaction between storytelling and ICH labels will be stronger in the gift-giving context than in the self-purchase context.	Supported
*H7*: Cultural identity mediates the effects of packaging cues on purchase intention.	Partially supported
*H7a*: Storytelling indirectly increases consumers’ purchase intention by enhancing cultural identity.	Supported
*H7b*: An ICH label indirectly increases consumers’ purchase intention by enhancing cultural identity.	Not supported
*H7c*: The interaction between storytelling and an ICH label indirectly increases consumers’ purchase intention by enhancing cultural identity.	Partially supported
*H8*: Purchase motivation moderates the indirect effects of packaging cues on purchase intention through cultural identity.	Not supported
*H8a*: The indirect effect of storytelling on purchase intention through cultural identity will be stronger in the self-purchase context than in the gift-giving context.	Not supported
*H8b*: Purchase motivation moderates the indirect effect of an ICH label on purchase intention through cultural identity.	Not supported
*H8c*: The indirect effect of the interaction between storytelling and an ICH label on purchase intention through cultural identity will be stronger in the gift-giving context than in the self-purchase context.	Not supported

## 5 Discussion

### 5.1 Theoretical findings

The central finding of this study is not that storytelling is generally superior to ICH labels, but rather that the effects of these two cultural cues depend on how they are combined and on the purchase context in which they are encountered. In the present study, storytelling significantly increased both cultural identity and purchase intention, whereas the ICH label did not produce a stable overall effect. However, in the gift-giving context, the joint presentation of storytelling and the ICH label generated a stronger positive effect. The conditional indirect-effect analysis further showed that storytelling was positively associated with purchase intention through cultural identity across different ICH label and purchase motivation conditions, whereas the indirect effect of the ICH label depended on whether storytelling was present. Although some conditional indirect effects varied across contexts, the moderating effect of purchase motivation on the overall indirect effects did not reach statistical significance. Taken together, these findings suggest that the effects of ICH-related packaging cues cannot be understood solely in terms of the average effect of any single cue; rather, the configuration of multiple cues and the purchase context in which they are used must be considered simultaneously.

Building on this finding, the present study first advances cue utilization theory by extending its explanation of the relationships among heterogeneous cultural cues. Prior research has largely focused on the average effects of individual narrative or certification cues, whereas the present findings show that storytelling and ICH labels are neither universally substitutable nor unconditionally complementary. Instead, the relative effects of different cultural cues and their combinations vary across purchase contexts, a pattern consistent with the notion of context-dependent cue diagnosticity (Barretta et al., 2025; Olson, 1972). In this sense, the study extends cue utilization research beyond the effects of individual cues to the configuration of multiple cues and the contextual dependence of their diagnostic value.

Second, this study extends the application of construal level theory from differences in attribute preferences between self and other-oriented decision-making to the selective utilization of combinations of packaging cues. Existing research has primarily shown that psychological distance alters consumers’ attention to concrete attributes versus abstract values (Trope and Liberman, 2010; Baskin et al., 2014). The present study found that storytelling alone was sufficient to generate positive effects in the self-purchase context, whereas the combination of storytelling and the ICH label produced stronger effects in the gift-giving context. This suggests that purchase motivation influences not only which product attributes consumers prioritize but also the relative diagnostic value of different cultural cues and their combinations in decision-making. The study therefore extends construal level theory to multi-cue packaging decisions.

Third, this study further clarifies the role of cultural identity in ICH consumption. Prior studies have typically treated cultural identity as an antecedent of purchase intention for ICH products, whereas the present study places cultural identity within the process through which specific cultural cues on packaging are processed and translated into purchase responses (Zhang et al., 2023). The findings indicate that cultural identity is not related to all packaging cues in the same manner. The indirect association generated by storytelling was relatively stable, whereas the indirect effect of the ICH label depended on how it was combined with storytelling. The study thus extends cultural identity research beyond a general identity orientation or purchase antecedent to consumers’ selective responses to specific forms of cultural communication.

More specifically, this study did not identify a stable moderated mediation mechanism involving purchase motivation. Although purchase motivation altered some relationships between packaging cues and cultural identity and also changed the strength of the association between cultural identity and purchase intention, these changes did not jointly produce significant differences in the overall indirect effects. In particular, within the gift-giving context, the effects of some cultural cues on cultural identity became stronger, whereas the association between cultural identity and purchase intention became comparatively weaker. This pattern suggests that purchase motivation may exert differentiated effects at different stages of the mediating process. One possible explanation is that gift-giving increases consumers’ attention to cultural information, making cultural cues more likely to foster cultural identity. At the same time, final purchase decisions in gift-giving contexts also involve considerations such as gift–recipient fit, anticipated recipient reactions, and social expression, which may reduce the relative contribution of cultural identity to purchase decisions. Consequently, a stronger first-stage relationship does not necessarily translate into a stronger overall indirect effect. The pattern observed here therefore appears to reflect differentiated effects of the purchase context across different stages of the cultural identity pathway, rather than a uniform strengthening or weakening of the entire mediation mechanism. Because the present study did not directly measure these gift-giving-related mechanisms, this explanation requires further empirical examination.

The relatively large effect of storytelling on purchase intention should also be interpreted with caution. This effect should be understood as reflecting the difference between the two specific packaging texts used in the present study and should not be generalized to all ICH food products or purchase contexts. In addition to its narrative structure, the storytelling text contained more specific historical, transmission-related, and cultural information. Its effect may therefore reflect both narrative processing and enhanced understanding of cultural information. Although the manipulation checks revealed no significant differences between the two text conditions across multiple potential confounding factors, the study did not directly assess participants’ awareness or guesses regarding the research purpose. The possible influence of experimental demand characteristics therefore cannot be completely ruled out.

Finally, it is noteworthy that the ICH label was successfully recognized by participants but did not produce a stable overall effect across contexts. This finding further indicates that the recognizability of a cultural certification cue does not necessarily imply stable diagnostic value in purchase judgments. Rather, the effectiveness of an ICH label needs to be understood in conjunction with the specific cue configuration in which it appears and the purchase context in which the decision is made.

### 5.2 Managerial implications

The findings of this study suggest that the configuration of cultural cues on ICH food packaging should be aligned with the specific purchase motivation, rather than simply combining storytelling and ICH labels indiscriminately. From the perspective of cue utilization theory, purchase motivation changes the diagnostic value of different cultural cues for the judgment at hand. The results further show that storytelling alone can generate positive effects in the self-purchase context, whereas the combination of storytelling and an ICH label produces stronger effects in the gift-giving context.

In the self-purchase context, firms may prioritize the use of concise cultural stories organized around product origins, transmission processes, production techniques, and local food-consumption contexts. Such content can help consumers develop a more concrete understanding of the cultural meanings embodied in the product. Prior research has likewise shown that information about product origin, production processes, and product stories may enhance consumers’ perceptions of the distinctive value of food products (Loebnitz et al., 2015; Wu and Lee, 2016; Youn and Kim, 2017).

In the gift-giving context, firms may combine storytelling with an ICH label. Storytelling can communicate the specific cultural content of the product, whereas the ICH label provides a concise and recognizable signal of cultural affiliation. Used together, these two cues allow the packaging to perform both cultural-explanatory and identity-signaling functions. This recommendation is consistent with the context-dependent cue-combination effect identified in the present study and with the theoretical view that gift-giving decisions place greater emphasis on overall value, symbolic meaning, and relational expression (Lyu et al., 2022; Baskin et al., 2014).

At the societal level, cultural authorities and ICH safeguarding organizations could establish mechanisms for compiling and reviewing story materials related to ICH food, with the participation of inheritors, local communities, and relevant experts. Such mechanisms could systematically document product origins, production techniques, consumption customs, and transmission histories, while clearly specifying the factual basis and permissible scope of use for these stories. On this basis, firms, cultural and tourism organizations, and public cultural institutions could appropriately adapt verified story materials for different contexts, such as guided interpretation, exhibitions, and cultural education. This approach may improve public understanding of the cultural meanings embodied in ICH food while reducing the risks of factual distortion and oversimplification of cultural significance during communication.

The evaluation of related public communication programs should not focus solely on product sales or communication exposure. It may also incorporate indicators such as cultural understanding, cultural identity, willingness to learn more, and willingness to share cultural information (Rashidin et al., 2026). This would make it possible to assess whether communication initiatives generate effects at both the cultural cognition and cultural identification levels, thereby providing a stronger basis for improving future public communication and safeguarding practices related to ICH food.

## 6 Conclusion

Based on an imagined online purchase scenario and focusing on a local Chinese ICH food, this study examined the effects of storytelling, ICH labels, and purchase motivation on consumers’ cultural identity and their purchase intention. The results showed that storytelling significantly enhanced both cultural identity and purchase intention, whereas the ICH label did not produce a stable independent effect. In the self-purchase context, storytelling alone generated positive effects, while in the gift-giving context, the joint presentation of storytelling and the ICH label produced stronger positive effects.

The conditional indirect-effect analysis further showed that storytelling was positively associated with purchase intention through cultural identity across different ICH label and purchase motivation conditions. In the gift-giving context, the direction of the indirect effect of the ICH label depended on whether storytelling was simultaneously present. However, the moderating effect of purchase motivation on the overall conditional indirect effects did not reach statistical significance. Because cultural identity and purchase intention were measured concurrently, these findings should be interpreted as conditional indirect associations consistent with the proposed theoretical mediation pathways rather than as evidence of an established causal mediation mechanism. Importantly, the ICH label itself did not produce a significant independent effect, indicating that its effectiveness depends on the specific narrative configuration and purchase context in which it is presented.

Overall, within the specific product, sample, and experimental context examined in this study, the findings clarify the role of cultural identity in linking cultural cues on ICH food packaging to purchase intention and identify the boundary conditions under which storytelling and ICH labels operate synergistically. These findings provide preliminary guidance for configuring cultural information on ICH food packaging, although their generalizability should be further examined across different products, more representative samples, and real purchase or willingness-to-pay contexts.

## 7 Limitations and future research

First, this study focused on a single ICH food and employed an imagined online purchase scenario. The findings may therefore have been influenced by product attractiveness, health perceptions, cultural relevance, and gift suitability. Although familiarity with official ICH labels was measured, the study did not directly assess participants’ knowledge of the certifying institution, trust in the label, perceptions of label visibility, or judgments of certification authenticity. Accordingly, the nonsignificant overall main effect of the ICH label may partly reflect limited prior trust or insufficient understanding of its meaning.

In addition, the study used relatively fixed packaging stimuli and text-based storytelling, which limits the generalizability of the findings across different cultural backgrounds, categories of ICH food, and packaging-design contexts. Future research could replicate the study using different types of ICH food, conventional traditional foods, and consumers from different regions and cultural backgrounds. It could also further examine how design elements such as visual imagery, color, typography, and packaging structure influence the perception of cultural cues and subsequent purchase decisions (Schröder et al., 2025).

Second, this study did not include research-purpose guessing items or suspicion probes and therefore could not directly determine whether participants inferred the study hypotheses. Although the focal variables were measured before the manipulation checks and no significant differences were found between the two text conditions across multiple potential confounding factors, the possibility that demand characteristics contributed to the relatively large effect size of storytelling cannot be completely ruled out. Future research could incorporate research-purpose guessing checks, use more subtle stimulus manipulations, and include behavioral measures such as actual product choice or willingness to pay to reduce demand characteristics and biases associated with self-reported measures.

Third, participants were recruited through convenience sampling on social media and snowball sampling, which may have introduced self-selection bias. Individuals who voluntarily completed the questionnaire may have been more attentive to local food and cultural information than consumers in general, potentially amplifying their responses to storytelling cues. Future research should consider stratified or quota sampling, include consumers with no prior experience purchasing local food, and employ behavioral measures such as actual product choice, field experiments, or willingness to pay to improve external validity.

Fourth, cultural identity and purchase intention were measured concurrently, meaning that the relationship between them remains correlational. Although the factorial conditional process analysis showed that the observed data were consistent with the proposed conditional indirect pathways, it cannot establish that cultural identity temporally precedes purchase intention, nor can it rule out reverse causality or common method bias. Future research should further validate the measurement of cultural identity across different products and cultural contexts and employ temporally separated measurements or alternative research designs to test the mediating relationship. Additional mechanisms, such as perceived authenticity, trust, and narrative transportation, also warrant examination.

Fifth, this study did not directly measure other psychological mechanisms associated with different purchase motivations. Participants in the gift-giving context may also have considered gift–recipient fit, symbolic value, anticipated recipient evaluations, and impression-management concerns, whereas participants in the self-purchase context may have placed greater emphasis on personal taste, utilitarian value, price, and individual preferences. Although the experiment standardized the recipient, relationship type, gift-giving occasion, budget conditions, and dietary restrictions, social-evaluation risk and gift–recipient fit risk could not be fully eliminated from the gift-giving context. Future research should measure these competing mechanisms simultaneously and compare their explanatory power with that of cultural identity (Rashidin et al., 2025).

## Data Availability

The raw data supporting the conclusions of this article will be made available by the authors, without undue reservation.
