# Supplementary material for: How packaging cues shape purchase intentions for intangible cultural heritage foods: evidence from heritage labels and storytelling
Source: Front Psychol. 2026 Sep 23;17:1930687. doi: 10.3389/fpsyg.2026.1930687 (PMC13643914; doi:10.3389/fpsyg.2026.1930687)
Supplement: Supplementary file 1 [file Supplementary_file_1.DOCX]

Supplementary Material S1: Main-Study Manipulation and Potential Confound Checks

After viewing the randomly assigned packaging image and completing the measures of the main variables, including cultural identity and purchase intention, please respond to the following items. 1 = strongly disagree, 2 = disagree, 3 = somewhat disagree, 4 = neither agree nor disagree, 5 = somewhat agree, 6 = agree, and 7 = strongly agree.

1 Text Manipulation

1.1 Perceived Narrativity

(1) The text on the packaging reads like a story.

□1 □2 □3 □4 □5 □6 □7

(2) The text on the packaging includes identifiable characters and events.

□1 □2 □3 □4 □5 □6 □7

(3) The text on the packaging presents events in a chronological or causal sequence.

□1 □2 □3 □4 □5 □6 □7

2 Potential Confounds in the Text Conditions

2.1 Perceived Informativeness

(1) The packaging provides sufficient product information.

□1 □2 □3 □4 □5 □6 □7

2.2 Text Readability

(1) The text on the packaging is clear and easy to read.

□1 □2 □3 □4 □5 □6 □7

2.3 Visual Salience

(1) The main information on the packaging is visually prominent and readily attracts my attention.

□1 □2 □3 □4 □5 □6 □7

2.4 Packaging Attractiveness

(1) The overall visual presentation of the packaging is attractive.

□1 □2 □3 □4 □5 □6 □7

2.5 Perceived Credibility of Packaging Information

(1) I believe that the information conveyed on the packaging is authentic.

□1 □2 □3 □4 □5 □6 □7

(2) I believe that the information conveyed on the packaging is credible.

□1 □2 □3 □4 □5 □6 □7

2.6 Perceived Emotionality

(1) The text on the packaging has a strong emotional tone.

□1 □2 □3 □4 □5 □6 □7

(2) The text on the packaging readily elicits an emotional response from me.

□1 □2 □3 □4 □5 □6 □7

2.7 Information Valence

(1) The text on the packaging describes the product positively.

□1 □2 □3 □4 □5 □6 □7

(2) The overall product information conveyed by the text on the packaging is positive.

□1 □2 □3 □4 □5 □6 □7

2.8 Perceived Cognitive Effort

(1) Understanding the text on the packaging requires considerable mental effort.

□1 □2 □3 □4 □5 □6 □7

（2）Processing the information conveyed by the text on the packaging is effortful for me.

□1 □2 □3 □4 □5 □6 □7

3 Label Manipulation

3.1 Label Recognition

(1) Did you see an intangible cultural heritage label on the packaging?

□ Yes □ No □ Unsure

3.2 Perceived Official Recognition

(1) The packaging leads me to believe that the product has received formal or official recognition as intangible cultural heritage.

□1 □2 □3 □4 □5 □6 □7

4 Purchase-Motive Manipulation

4.1 Perceived Self-Purchase Motive

(1) I would purchase this product primarily for my own consumption.

□1 □2 □3 □4 □5 □6 □7

4.2 Perceived Gift-Purchase Motive

(1) I would purchase this product primarily as a gift for others.

□1 □2 □3 □4 □5 □6 □7
